# Experience-Dependent Specialization of Receptive Field Surround for Selective Coding of Natural Scenes

**DOI:** 10.1016/j.neuron.2014.09.010

**Published:** 2014-10-22

**Authors:** Michael Pecka, Yunyun Han, Elie Sader, Thomas D. Mrsic-Flogel

**Affiliations:** 1 Department of Neuroscience, Physiology, and Pharmacology, University College London, 21 University Street, London WC1E 6DE, UK; 2 Biozentrum, University of Basel, Klingelbergstrasse 50/70, 4056 Basel, Switzerland

## Abstract

At eye opening, neurons in primary visual cortex (V1) are selective for stimulus features, but circuits continue to refine in an experience-dependent manner for some weeks thereafter. How these changes contribute to the coding of visual features embedded in complex natural scenes remains unknown. Here we show that normal visual experience after eye opening is required for V1 neurons to develop a sensitivity for the statistical structure of natural stimuli extending beyond the boundaries of their receptive fields (RFs), which leads to improvements in coding efficiency for full-field natural scenes (increased selectivity and information rate). These improvements are mediated by an experience-dependent increase in the effectiveness of natural surround stimuli to hyperpolarize the membrane potential specifically during RF-stimulus epochs triggering action potentials. We suggest that neural circuits underlying surround modulation are shaped by the statistical structure of visual input, which leads to more selective coding of features in natural scenes.

## Highlights

• V1 firing is more selective to natural than phase-scrambled surround stimuli
• Improved selectivity is caused by Vm hyperpolarisation prior to RF-spiking events
• Natural surround sensitivity is experience dependent, absent in immature/deprived V1
• Vision shapes circuits to improve V1 coding of natural scenes across RF and surround

## Teaser

Pecka et al. show that surround modulation in primary visual cortex is tuned during development so that neurons become more selective for features embedded in full-field natural scenes.

## Introduction

The visual system is specialized to extract features from complex natural scenes that have a unique statistical structure (Simoncelli and Olshausen, 2001, Felsen et al., 2005a), including edges and contours that change in space and time across the field of view. Although neurons in the primary visual cortex (V1) respond best to local image features that fall within their receptive fields (RFs), their responses are strongly modulated by stimuli placed in the surrounding regions of visual space (Blakemore and Tobin, 1972, Nelson and Frost, 1978, Allman et al., 1985, Gilbert and Wiesel, 1990). Typically, stimulating the surround suppresses responses to stimuli in the RF (Jones et al., 2001, Jones et al., 2002, Seriès et al., 2003, Ozeki et al., 2009, Adesnik et al., 2012), and this suppression is more pronounced when using natural surround images than when using their phase-scrambled versions devoid of complex structure (Guo et al., 2005). Visual circuits are thus particularly sensitive to integrating salient image components across natural scenes, which may contribute to contour integration and “pop-out” phenomena at the perceptual level (Knierim and van Essen, 1992). Concomitantly, surround modulation by natural images alters the firing rate distribution of individual neurons, whereby their responses become more selective and sparse (Vinje and Gallant, 2000, Vinje and Gallant, 2002, Haider et al., 2010). Sparse codes are considered efficient, because they are able to transfer more information with fewer spikes (Olshausen and Field, 2004). Taken together, surround modulation contributes to contextual processing of sensory information and increases the efficiency of neural representations for natural scenes (Sachdev et al., 2012).

How do neural circuits become specialized to integrate and efficiently represent information from complex natural scenes, which contain image features that extend beyond the RF of any individual neuron? Neurons in V1 are feature selective already at eye opening (Hubel and Wiesel, 1963, Blakemore and Van Sluyters, 1975, Chapman and Stryker, 1993, Krug et al., 2001, White et al., 2001, Rochefort et al., 2011, Ko et al., 2013). However, little is known about the development of surround modulation and its dependence on early sensory experience, and how this impacts the ability to encode complex natural scenes. Surround modulation is mediated by excitatory and inhibitory interactions at different stages of the mature visual pathway, including the retina (Olveczky et al., 2003, Solomon et al., 2006) and visual cortex (Stettler et al., 2002, Angelucci and Bressloff, 2006, Girardin and Martin, 2009, Ozeki et al., 2009, Haider et al., 2010, Adesnik et al., 2012, Nienborg et al., 2013, Vaiceliunaite et al., 2013). Since both excitatory and inhibitory circuits refine after eye opening (Frégnac and Imbert, 1984, Katagiri et al., 2007, Kuhlman et al., 2011, Ko et al., 2013) and are susceptible to changes in visual experience (Ruthazer and Stryker, 1996, Zufferey et al., 1999, White et al., 2001, Chattopadhyaya et al., 2004, Maffei et al., 2004), the effectiveness of surround modulation may be expected to change during postnatal development. The extent to which this may improve the processing of full field natural scenes is, however, unknown.

In this study, we show that circuits mediating surround modulation require sensory experience to become preferentially sensitive to natural stimulus statistics across the RF and its surround. In mature mouse V1, neuronal firing to natural movies presented to the RF became more selective when costimulating the surround with natural movies than with phase-scrambled movies lacking the higher-order statistical regularities of natural scenes. In contrast, this preferential sensitivity of center-surround interactions for natural scenes was absent in immature, visually naive V1 after eye opening and in mature animals that were reared without visual input. Mechanistically, the surround-induced increase of response selectivity was mediated by transient membrane potential hyperpolarization that coincided with moments of greatest depolarization during RF stimulation. These transient hyperpolarizing events were most effective in limiting spiking during full-field natural movie stimulation in adult V1, consistent with the increased effectiveness of the natural surround stimuli in improving response selectivity. Therefore, normal visual experience is required for the refinement of neuronal circuits that contribute to the selective coding of natural scenes by spatially integrating information from the entire field of view.

## Results

### Surround Suppression in Mature and Developing Mouse V1

**Figure 1:**
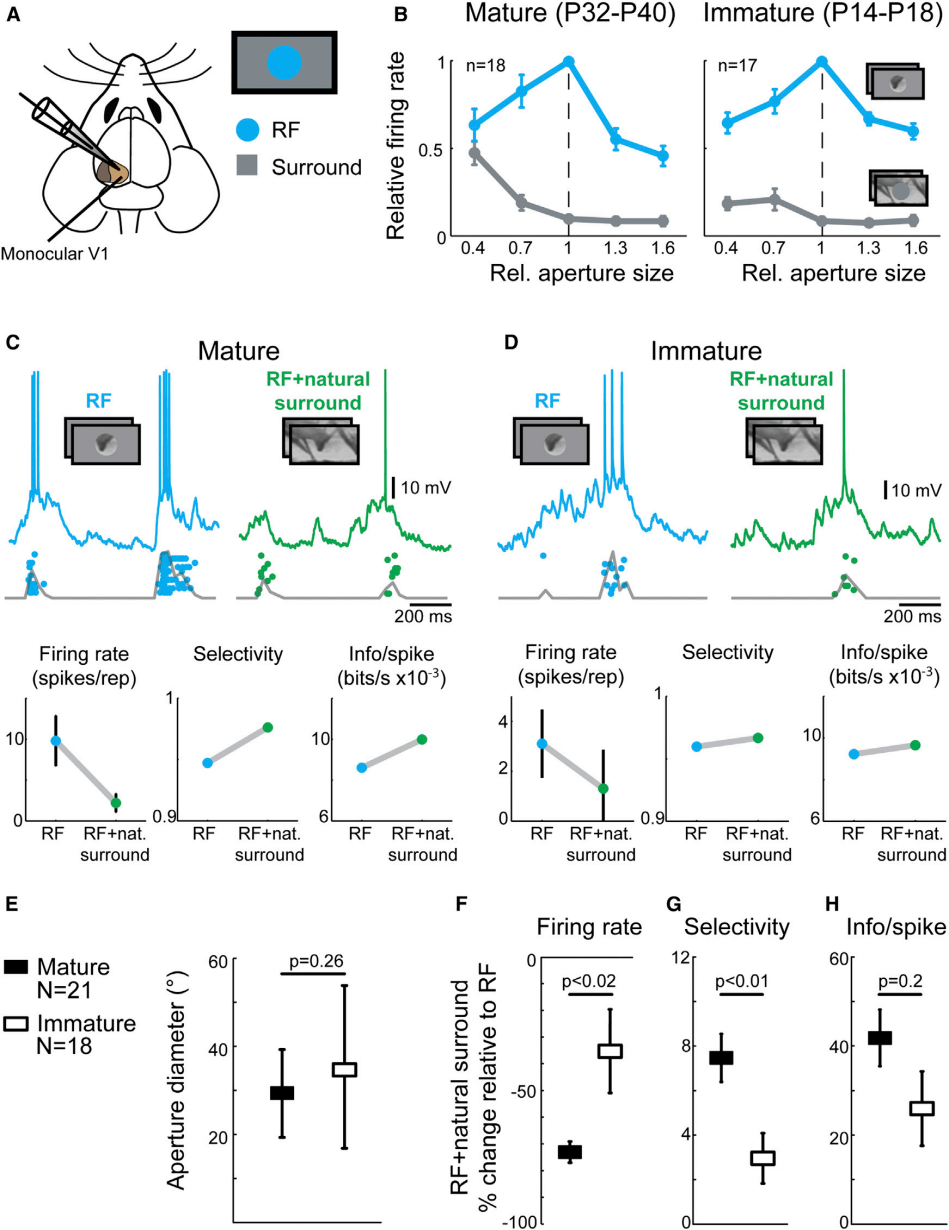
Surround-Induced Increases in Response Suppression and Selectivity Are Present in Immature V1 but Are More Pronounced in Mature V1 (A) Schematic depicting whole-cell patch-clamp recordings of neurons in the monocular region of primary visual cortex (V1) of anesthetized mice. The size of the receptive field (RF) and the influence of the surround were determined by presentation of a naturalistic movie to the contralateral eye, by varying the size of the center (aperture) and surrounding (annulus) stimuli. (B) Averaged normalized size-tuning functions of V1 neurons in mature and immature mice. The mean normalized firing rates (±SEM) at aperture sizes relative to optimal RF size (“1”) are shown for apertured (RF only) and corresponding annulus (surround only) stimuli. Only neurons with similar increments in aperture size were included for this analysis (mature, n = 18; immature, n = 17). (C) Example whole-cell recording from a neuron in mature V1 during stimulation of RF or RF + natural surround. Voltage traces of a single repetition are shown with spike-dot-raster and spike-histogram of ten repetitions overlaid underneath the trace. Lower panels show metrics derived from this example recording, including mean firing rate (±SEM, left panel), selectivity index (middle panel), and mutual information/spike (right panel). (D) Example whole-cell recording from a neuron in immature V1 (postnatal day 18) during stimulation of the RF and RF + natural surround. Conventions as in (C). (E) Mean aperture (RF) diameter (±SEM) for all neurons recorded in mature (29.9° ± 10°; n = 21) and immature mice (35.3° ± 18°, n = 18) was not significantly different (p = 0.26, t test). (F–H) Mean population changes (%± SEM) in firing rate (F), selectivity (G), and mutual information/spike (H) during RF + natural surround stimulation normalized to RF stimulation for mature (black symbols) and immature (white symbols) mice. p values refer to differences in mean change across age groups (t test).

To study the effectiveness of surround modulation during postnatal development, we carried out in vivo whole-cell recordings from individual neurons in cortical layer 2/3 of monocular V1 in immature mice with limited visual experience (1–5 days after eye opening, P14–P19, n = 18 from 7 mice) and in visually mature mice with at least 18 days of normal visual experience (P32–P40, n = 21 from 10 mice). To determine the exact RF size of each recorded neuron, we alternated the presentation of a naturalistic movie within apertures of increasing size (isoluminant gray surround) and the corresponding surround (annulus) regions (Figure 1A; see Experimental Procedures). In both mature and immature V1, neuronal firing was stimulus size dependent (Figure 1B). Responses first increased and then decreased with increasing aperture size, while response rate decreased for the corresponding surround stimuli (Figure 1B, see figure legend for details). The RF size—defined by the aperture diameter at which neurons exhibited a maximal response without a significant response to the corresponding annulus stimulus—was similar for the two age groups (Figure 1E; mean ± SEM, mature, 29.9° ± 10°; immature, 35.3° ± 18°, p = 0.26, t test). While responses decreased significantly during full-field stimulation with natural movies (RF + natural surround; Figures 1C and 1D) compared to stimulation of the RF alone (p < 0.01 for both mature and immature mice, paired t test), they were suppressed more in mature V1 (Figure 1F, mature, −71.9% ± 3.6%; immature, −35.3% ± 15.6%, p = 0.019, t test). These results show that neurons in immature V1 exhibit surround suppression within a few days after eye opening, but that the suppressive effect of the surround becomes stronger with age.

To determine how the developmental strengthening of surround suppression influences the selectivity of neuronal responses, we computed a measure that captures the distribution of spiking across stimulus repetitions (here referred to as “selectivity,” also known as “lifetime sparseness”; Vinje and Gallant, 2002, Lehky et al., 2005, Tolhurst et al., 2009, Willmore et al., 2011; see Experimental Procedures). In both immature and mature V1, response selectivity increased significantly during natural surround stimulation compared to stimulation of the RF alone (Figure 1G; p < 0.01, paired t test), and this increase was significantly greater in mature animals (mature, 7.5% ± 1.1%; immature, 3.0% ± 1.1%, p = 0.008, t test).

A reduced spike rate and increased selectivity only add to the efficiency of a neuronal representation if the information about the stimulus is adequately maintained (Laughlin, 2001, Vinje and Gallant, 2002). Hence, the amount of information per spike should increase to compensate for fewer evoked spikes. In both age groups, costimulating the surround significantly increased the information per spike (see Experimental Procedures) relative to the stimulation confined to the RF (Figure 1H, p < 0.01, paired t test). This increase tended to be higher in mature than in immature V1 (mature, 41.9% ± 6.3%; immature, 26.2% ± 8.2%, p = 0.2, t test), but the effect did not reach significance. Very similar results were obtained in a separate data set using juxtacellular single-cell recordings (Figure S1 available online), indicating that any alterations of the intracellular milieu caused by the whole-cell recording technique did not influence the results. These age-dependent effects of the surround on firing rate suppression were not influenced by any differences in RF size or absolute firing rate between of neurons recorded in the two age groups (Figure S2). Taken together, these data indicate that visual circuits are capable of spatial integration already at eye opening, but that surround modulation becomes more effective at suppressing firing and increasing response selectivity to natural scenes with age.

### Natural Surround Increases Response Selectivity More Than Artificial Surround in Mouse V1

**Figure 2:**
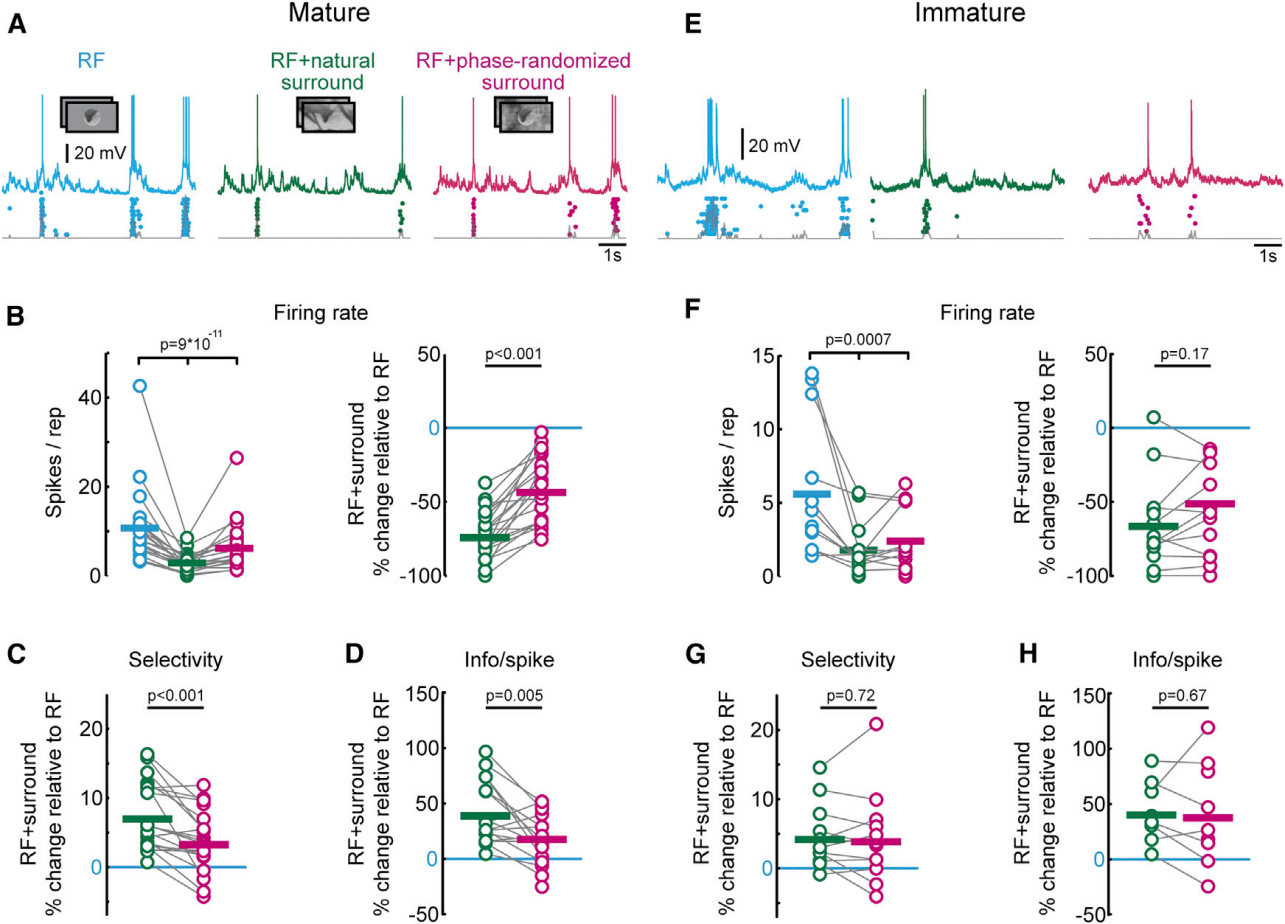
Neurons Are Sensitive to Natural Stimulus Statistics in the RF Surround in Mature, but Not in Immature, V1 (A) Example whole-cell recording from a layer 2/3 neuron in V1 of a mature mouse (postnatal day 36) during stimulation of the RF (left panel, blue trace), RF + natural surround (middle panel, green trace), and RF + phase-randomized surround (right panel, magenta trace). Conventions as in Figure 1C. (B) Firing rates during the three stimulus conditions (left panel), and changes (%) in firing rate (right panel) during stimulation of the RF + natural surround (green) or RF + phase-randomized surround (magenta) relative to stimulating the RF center in mature mice. Mean firing rates for each cell are shown by connected open circles. Horizontal bars denote group mean values. (C and D) Changes (%) in selectivity (C) and information/spike (D) during stimulation of the RF + natural surround (green) or RF + phase-randomized surround (magenta) relative to stimulating the RF center in mature mice (n = 21 cells; p values, paired t test). (E) Example whole-cell recording from a L2/3 neuron in immature V1 (postnatal day 16). Conventions as in (A). (F) Firing rate during the three stimulus conditions (left panel), and changes (%) in firing rate (right panel) during stimulation of the RF + natural surround (green) or RF + phase-randomized surround (magenta) relative to stimulating the RF center in immature mice (n = 13 cells). (G and H) Changes (%) in selectivity (G) and information/spike (H) during stimulation of the RF + natural surround (green) or RF + phase-randomized surround (magenta) relative to stimulating the RF center in immature mice. (n = 13 cells; p values, paired t test).

In adult monkey V1, the effectiveness of surround modulation depends on the higher-order structure of natural scenes (e.g., extended contours), because responses to natural images in the RF are suppressed less when randomizing the phase of natural images in the surround (Guo et al., 2005). We therefore tested whether neurons in mature mouse V1 also exhibit the dependency of RF-surround interactions on the statistical properties of surround stimuli. We compared how responses to the natural movie presented in the RF were altered by costimulating the surround either with the same natural movie (RF + natural surround) or with the phase-randomized version of the same movie (RF + phase-randomized surround, Figure 2A). Note that the phase randomization only removes the higher-order structure in natural images without altering their contrast or spatial frequency composition (see Experimental Procedures). Accordingly, full-screen presentations of natural and phase-randomized stimuli evoked similar activity levels in both age groups (Figure S3). For the following analysis, we only included neurons whose responses were significantly suppressed in at least one of the surround conditions (mature, 21/21 cells; immature, 13/18 cells; see Experimental Procedures). In mature V1, costimulation of the surround with both natural and phase-randomized stimuli reduced firing rates significantly (Figure 2B; p = 9 × 10^−11^, one-way ANOVA), increased response selectivity (Figure 2C; RF + natural surround, 7.5% ± 1.1%, p < 0.001; RF + phase-randomized surround, 3.7% ± 0.9%, p < 0.001; t test) and mutual information per spike (Figure 2D; RF + natural surround, 41.8% ± 7.4%, p < 0.001; RF + phase-randomized surround, 20.6% ± 6.2%, p < 0.001; t test) compared to stimulation of the RF alone. Importantly, however, stimulating the surround with natural movies decreased firing rates significantly more than phase-randomized surround movies (Figure 2B; p < 0.001, paired t test). This led to significantly greater increases in both selectivity and mutual information per spike during natural compared to phase-randomized surround stimulation (Figures 2C and 2D; p < 0.001 and p = 0.005, respectively, paired t test). Thus, neurons in mature V1 are sensitive to the higher-order regularities of natural stimuli extending beyond the RF boundary, which makes their responses more selective and informationally efficient.

### Preference for Natural Surround Stimuli Emerges during Development

We next determined whether the increased sensitivity of V1 neurons for natural surround stimuli is already apparent within a few days after eye opening. In immature mice, the costimulation of the RF with either natural or phase-randomized surround stimuli generated significant spike rate suppression (Figures 2E and 2F, p = 0.0007, one-way ANOVA), increased response selectivity (Figure 2G, natural surround, 4.7% ± 1.3%, p < 0.001; phase-randomized surround, 4.3% ± 1.8%, p < 0.001; t test), and information transmitted per spike (Figure 2H, natural surround, 43.2% ± 7.8%, p < 0.001; phase-randomized surround, 40.7% ± 12.8%, p < 0.001; t test). However, neither the amount of response suppression nor the increase in response selectivity and information per spike was significantly different between the two types of surround stimuli (Figures 2F–2H; p = 0.17, p = 0.72 and p = 0.67, respectively; paired t test). Thus, in contrast to experienced animals, neurons in immature V1 did not differentiate between naturalistic and phase-scrambled stimuli in the surround, suggesting that early circuits mediating surround modulation are not yet preferentially sensitive for higher-order structure of natural scenes extending beyond the RF.

### Selective Hyperpolarization during Center-Surround Interactions Leads to Greatest Response Suppression to Full-Field Natural Scenes

**Figure 3:**
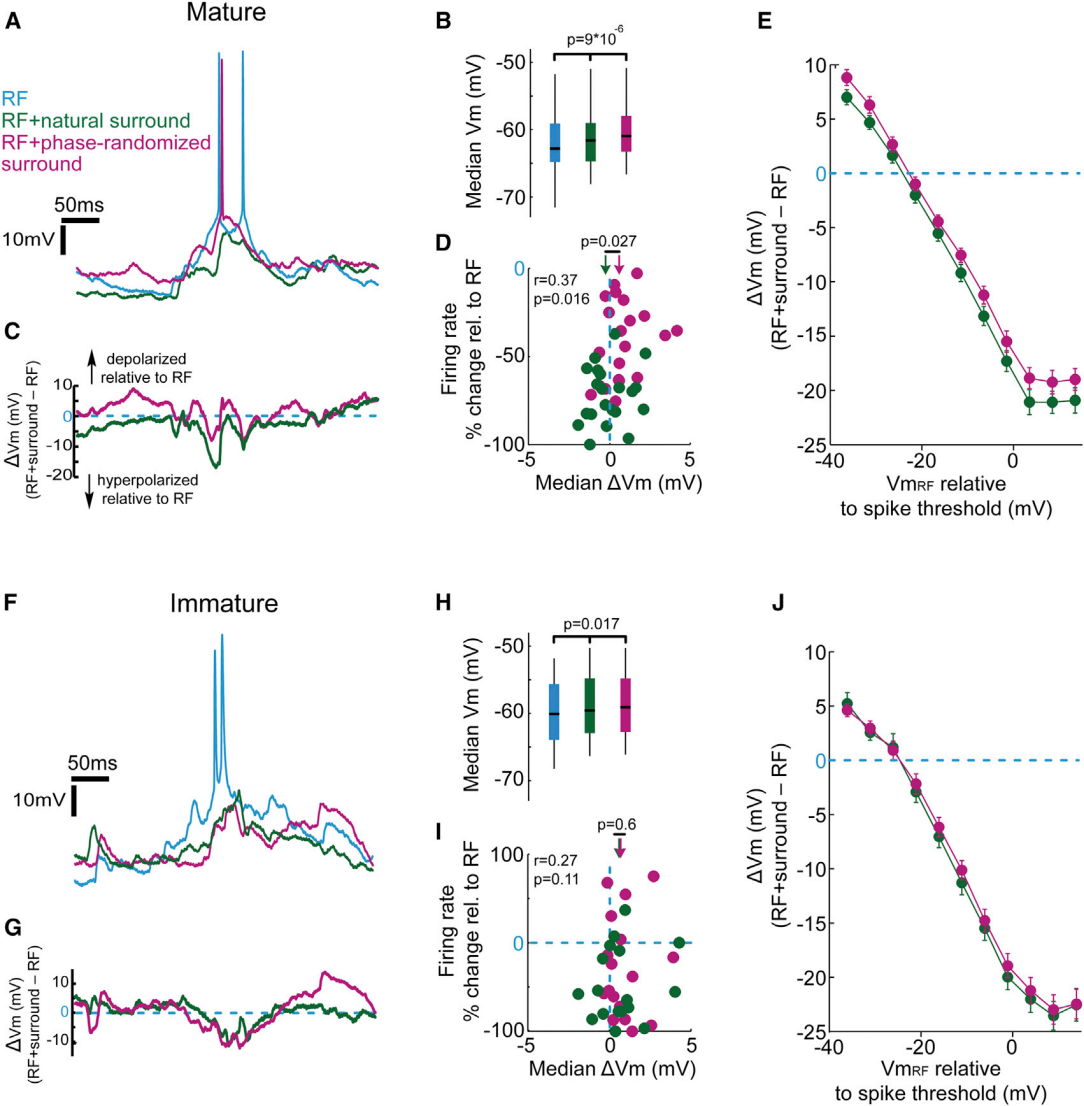
Natural and Phase-Randomized Surround Stimulation Elicits Significantly Different Hyperpolarization during RF Spiking Events in Mature, but Not Immature, V1 (A) Example recording from a L2/3 neuron in mature V1 (postnatal day 36) during presentation of the same movie sequence confined to the RF (blue) or when costimulating the surround with natural (green) or phase-randomized (magenta) movie. (B) The median Vm of mature V1 neurons (n = 21) during stimulation of the RF (blue), RF + natural surround (green) and RF + phase-randomized surround (magenta) was significantly different (RF, −62.8 mV; RF + natural surround, −61.6 mV; RF + phase-randomized surround, −60.9 mV; p = 9 × 10^−6^; Friedman’s test). Black mark inside colored box denote median values, while the box edges denote the 25th and 75th percentiles and the whiskers extending to the most extreme data points. (C) ΔVm obtained by subtracting the example traces in (A) after removal of spikes. (D) Median ΔVm during RF + natural surround stimulation (green) was significantly more negative than during RF + phase-randomized surround stimulation (magenta) (arrows denote population medians, p = 0.027, Wilcoxon sign-rank test). Firing rate suppression was strongly correlated with the median ΔVm (r = 0.37, p = 0.016). (E) Average ΔVm (±SEM) as a function of the Vm_RF_ (bin size 5 mV). ΔVm values in each bin were averaged after normalizing to the spike threshold for each cell. (F) Example recording from a L2/3 neuron in immature V1 (postnatal day 16) during presentation of the same movie sequence confined to the RF (blue) or when costimulating the surround with natural (green) or phase-randomized (magenta) movie. (G) ΔVm obtained by subtracting the example traces in (F) after removal of spikes. (H) The median Vm of immature V1 neurons (n = 18) during stimulation of the RF (blue), RF + natural surround (green) and RF + phase-randomized surround (magenta) was significantly different (RF, –60.1 mV; RF + natural surround, –59.6 mV; RF + phase-randomized surround, –59.1 mV; p = 0.017; Friedman’s test). (I) The median ΔVm during RF + natural surround stimulation (green) and during RF + phase-randomized surround stimulation (magenta) was not significantly different (population medians indicated by arrows, p = 0.6, Wilcoxon sign-rank test). Firing rate suppression was not correlated with the mean ΔVm (r = 0.27, p = 0.11). (J) Average ΔVm as a function of the Vm_RF_ (bin size 5 mV). ΔVm values in each bin were averaged after normalizing to the spike threshold for each cell in immature mice.

We next investigated whether the age-dependent increase in the sensitivity of center-surround interactions for natural scenes can be explained by differences in subthreshold membrane potential dynamics during different stimulus conditions (Figures 3A and 3F). Previous reports indicate that surround stimulation leads to more hyperpolarized membrane potential (Vm) relative to RF stimulation alone (Haider et al., 2010, Haider et al., 2013), which is partly attributed to increased inhibition in the cortical network (Haider et al., 2010, Adesnik et al., 2012, Nienborg et al., 2013, Vaiceliunaite et al., 2013). However, when averaged over the entire stimulation period, we found that costimulation of the surround with either natural or phase-scrambled movies slightly depolarized the median absolute Vm in immature and mature mice (Figures 3B and 3H; p = 0.017 and p < 0.0001, respectively; Friedman’s test).

Because it is unclear how such small average differences in Vm could contribute to changes in the spiking response selectivity, we focused our analysis on how Vm temporal dynamics are altered by surround stimulation. We quantified moment-to-moment differences in Vm between RF and full-field stimulation for each neuron (ΔVm = Vm_RF+surround_ − Vm_RF_; see Experimental Procedures). Both natural and phase-randomized surround stimuli induced hyperpolarizing (negative ΔVm) and depolarizing (positive ΔVm) Vm changes relative to RF stimulation alone (Figures 3C and 3G). Plotting the median ΔVm of each cell against its average change in firing rate revealed that ΔVm was strongly correlated with the firing rate suppression during full-field stimulation in mature, but not in immature mice (Figures 3D and 3I; see figure legend for details). Moreover, the distribution of ΔVm was shifted to more negative values during natural than phase-randomized surround stimulation in mature V1 (Figure 3D, p = 0.027, Wilcoxon rank sum test), but not in immature V1 (Figure 3I, p = 0.6, Wilcoxon rank sum test).

How could relatively small differences in ΔVm between natural and phase-randomized surround stimulation lead to pronounced differences in firing rate suppression incurred by these surround stimuli in mature V1? To address this question, we determined the dependency of ΔVm on the particular membrane potential value (relative to spike threshold) elicited by the RF stimulus at each time point during movie presentation (Vm_RF_). Strikingly, in both age groups, ΔVm exhibited a negative linear dependency on membrane depolarization during RF stimulation: neurons were relatively most hyperpolarized during RF + surround stimulation (negative ΔVm) specifically at times when Vm_RF_ was closest to spiking threshold (Figures 3E and 3J).

### GABAergic Inhibition Contributes to Selective Hyperpolarization during Surround Stimulation

**Figure 4:**
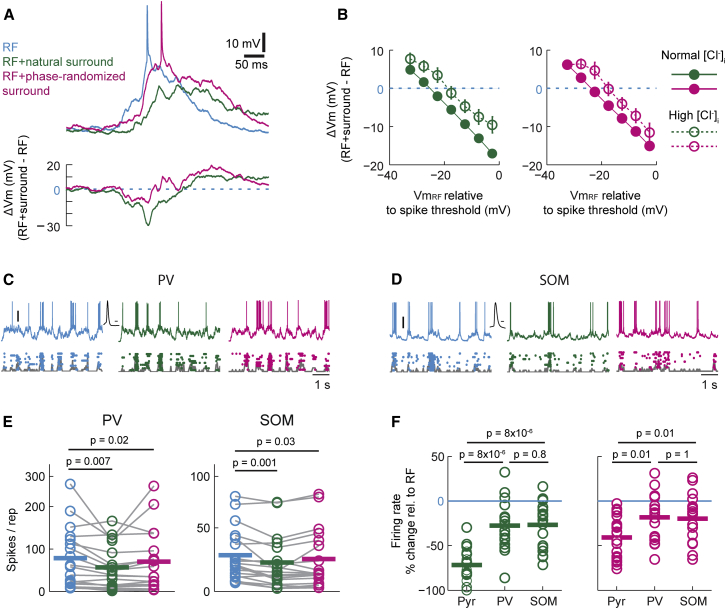
GABAergic Inhibition Contributes to Dependency of ΔVm on Vm_RF_ (A) Example recording with elevated [Cl^−^] of the internal solution from a V1 neuron in a mature mouse during presentation of the same movie sequence confined to the RF (blue) or during costimulation of the surround with natural (green) or phase-randomized (magenta) movies. Lower panel shows the ΔVm for the two surround conditions. (B) Elevated [Cl^−^] (open circles, n = 7) causes a rightward shift in the relationship between ΔVm and Vm_RF_ over the entire data range for both RF + natural (green) and RF + phase-randomized surround (magenta) conditions. See text for a detailed description. Data with normal [Cl^−^] are replotted from Figure 3E. (C) Example targeted whole-cell recording from a parvalbumin (PV)-positive interneuron in V1 from a mature PV-GFP mouse during stimulation of the RF (left panel, blue trace), RF + natural surround (middle panel, green trace), and RF + phase-randomized surround (right panel, magenta trace). Conventions as in Figure 2A. Scale bar, 20 mV. The black trace shows the action potential waveform of the example cell. Scale bar, 1 ms. (D) Example targeted whole-cell recording from a somatostatin (SOM) positive interneuron in V1 from a mature GIN mouse. Conventions as in (C). (E) Firing rates of individual parvalbumin (PV, n = 18) and somatostatin (SOM, n = 18) interneurons during stimulation of the RF center (blue), RF + natural surround (green) or RF + phase-randomized surround (magenta) in mature mice. Surround stimulation resulted in slight but significant decreases in firing rates (p values of paired comparisons given in the panel, Wilcoxon signed rank test). Conventions as in Figure 2. (F) Comparison of relative firing rates (% of firing rate during RF stimulation) of putative pyramidal cells (Pyr, n = 21), PV (n = 18), and SOM (n = 18) interneurons during costimulation of the surround with either RF + natural (green, left panel) or RF + phase-randomized (magenta, right panel) movies. PV and SOM neurons were significantly less suppressed during either surround condition compared to Pyr cells (p values of comparisons given in the panel, Mann-Whitney U test).

Which mechanisms underlie the pronounced surround-induced relative hyperpolarization when the Vm is most depolarized during RF stimulation? Surround stimulation has been shown to increase synaptic inhibition (Haider et al., 2010, Haider et al., 2013, Adesnik et al., 2012). We therefore tested the influence of chloride (Cl^−^)-mediated conductances on the inverse relationship between ΔVm and Vm_RF_. We performed whole-cell recordings using an elevated Cl^−^ concentration in the intracellular solution ([Cl^−^]_i_, see Experimental Procedures) to modify the reversal potential of GABA_A_-mediated conductances (Figure 4A). Compared to data recorded with the normal Cl^−^-concentration (Figure 3E), recordings with elevated [Cl^−^]_i_ revealed a rightward shift of ΔVm values in both natural and phase-randomized surround conditions at all values of Vm_RF_ (Figure 4B). These data suggest that an increased Cl^−^ conductance during surround stimulation at least in part contributes to the negative relationship of ΔVm and Vm_RF_. The Cl^−^ conductance may also account for the depolarizing effect of surround costimulation (positive ΔVm values, Figure 4B) at very hyperpolarized levels of Vm_RF_, if these fall below the reversal potential for GABA_A_-mediated conductances.

Given that the increased Cl^−^ conductance is most likely mediated by GABA_A_ receptors, we explored the likely sources of GABAergic inputs by targeting parvalbumin (PV) and somatostatin (SOM) inhibitory interneurons with whole-cell recordings (Figures 4C and 4D; see Experimental Procedures). We found that firing rates of PV and SOM neurons were on average only slightly but significantly reduced by costimulation of the surround relative to stimulation of the RF alone, irrespective of the surround stimulus type (Figure 4E).The relative firing rate decrease was smaller in both PV and SOM cells compared to putative pyramidal (Pyr) cells during either surround stimulus condition (Figure 4F, RF + natural surround, Pyr-PV p = 8 × 10^−6^; Pyr-SOM p = 8 × 10^−6^; RF + phase-scrambled surround, Pyr-PV p = 0.01; Pyr-SOM p = 0.01, Mann-Whitney U test). Since PV and SOM cells maintained relatively high firing rates during RF + surround stimulation, both interneuron classes can be expected to contribute to the increased inhibition of Pyr cells during surround stimulation.

Importantly, neither PV nor SOM cells were preferentially selective for the natural and phase-randomized surround stimuli (Figure 4F, PV p = 0.12 and SOM p = 0.14, for RF + natural versus RF + phase-randomized surround, paired t test). This is consistent with the observation that the rightward shift of the ΔVm and Vm_RF_ relationship after elevating [Cl^−^]_i_ was not associated with a change in the slope of the relationship (Figure 4B), suggesting that surround stimuli of different statistics cause no major difference in the average increase of Cl^−^ conductance. These data further imply an additional involvement of other, most likely excitatory conductances, suggesting that surround suppression is rooted in the modulation of temporally balanced excitation and inhibition (Ozeki et al., 2009).

### Surround-Induced Hyperpolarization at Times of Spike Generation during RF Stimulation

**Figure 5:**
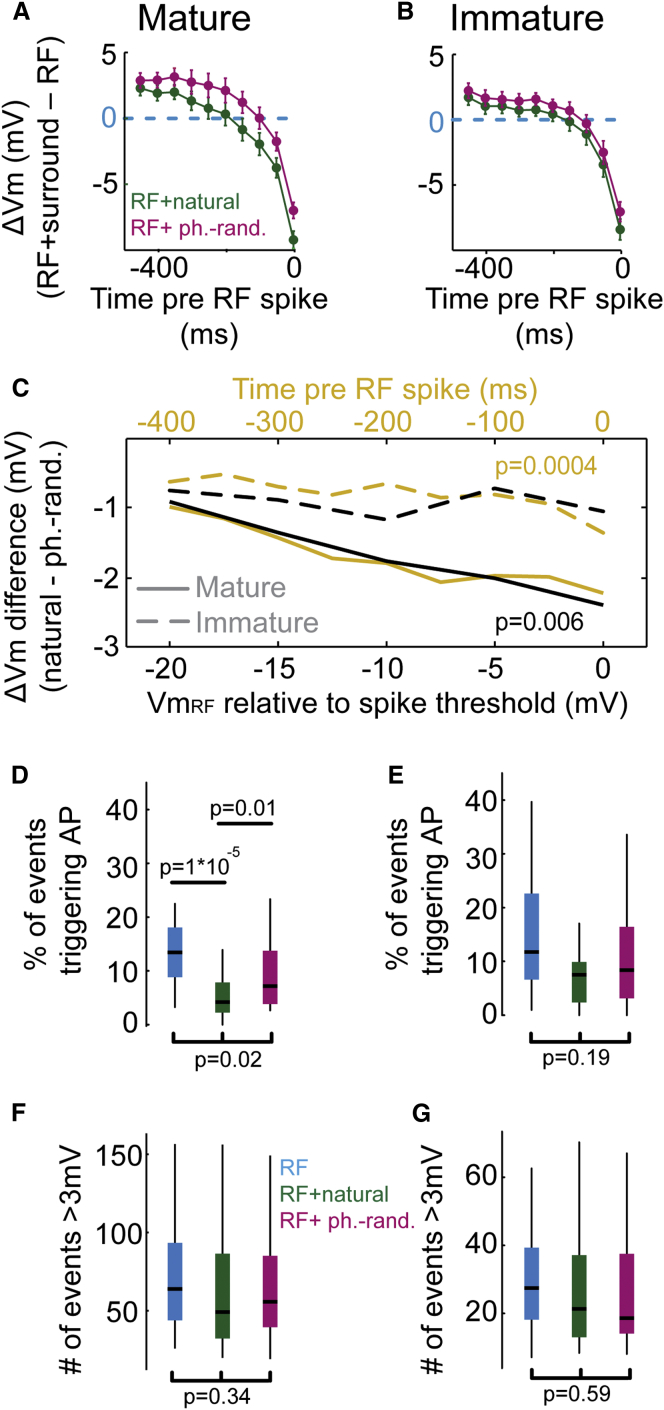
Precisely Timed Hyperpolarization Prior to RF Spiking Events Mediates Selective Surround Suppression (A) ΔVm as a function of the time before a spike during RF stimulation in mature mice. The mean ΔVm (±SEM) is plotted for the corresponding times (bin = 50 ms) during RF + natural (green) and RF + phase-randomized surround (magenta) conditions. ΔVm was significantly different between the surround conditions (F_interaction_ = 143, p < 0.00001, two-way ANOVA). (B) ΔVm as a function of the time before a spike during RF stimulation in immature mice. ΔVm was significantly different between the surround conditions (F_interaction_ = 103, p < 0.00001, two-way ANOVA). (C) Quantification of differences in ΔVm between RF + natural and RF + phase-randomized surround conditions in mature (solid lines) and immature (dashed lines) mice. Differences in ΔVm between surround conditions were consistently larger for mature mice, both when analyzed relative to Vm_RF_ (black, compare Figures 3E and 3J) and relative to time of RF spike (yellow, compare A and B). Note that only in mature mice differences in ΔVm between RF + natural and RF + phase-randomized surround conditions were large at times prior RF spiking (yellow solid line, p = 0.0004, t test) and increased with increasing depolarization of Vm_RF_ (black solid line, p = 0.006, t test). Thus, these differences in ΔVm might underlie the increased firing rate suppression during RF + natural surround stimulation. (D–G) Same conventions as in Figure 3B. (D) In mature V1, natural surround stimulation reduced the likelihood for large-amplitude synaptic events to trigger a spike in mature V1 (RF versus RF + natural surround, p = 1 × 10^−5^; RF + natural surround versus RF + phase-randomized surround, p = 0.01; Mann-Whitney U test). (E) In immature V1, the likelihood for large-amplitude synaptic events to trigger a spike was not significantly different across conditions (p = 0.19, Kruskal-Wallis test across all three conditions). (F) In mature V1, large-amplitude, depolarizing synaptic events were similarly frequent across stimulation conditions (p = 0.34; Kruskal-Wallis test across all three conditions). (G) In immature V1, large-amplitude, depolarizing synaptic events were similarly frequent across stimulation conditions (p = 0.59; Kruskal-Wallis test across all three conditions).

Our results thus far suggest that the increased response suppression and selectivity of putative pyramidal neurons during RF + natural surround stimulation in mature mice could not be explained by a net difference in the amount of inhibition during the two surround conditions. Instead, our results raise the possibility that the timing of inhibition may be important for this selective suppression, because the difference in ΔVm between natural and phase-randomized surround stimulation was largest at most depolarized Vm_RF_ (i.e., closest to spike threshold) in mature mice (Figure 5C; see also Figure 3E). To examine the timing of surround-induced hyperpolarization in more detail, we determined the temporal progression of ΔVm before the occurrence of a spike during RF stimulation (see Experimental Procedures). At times preceding action potential firing events during RF stimulation (corresponding to instances when the Vm is most depolarized, Figure 5C), natural surround stimuli hyperpolarized the Vm more than phase-randomized surround stimuli (Figures 5A, 5C, and S4D). This difference in the relative hyperpolarization between natural and phase-randomized surround (ΔVm difference) was significantly larger in mature mice compared to immature mice (Figures 5A–5C and S4H), both when ΔVm was binned relative to Vm_RF_ (p = 0.006, t test) and relative to RF spike time (p = 0.0004, t test). These findings are consistent with the greatest spike rate suppression during natural surround stimulation in mature V1 (Figures 2B and 3D), and suggest that suppression is caused by time-locked Vm hyperpolarization that curtails spike generation at moments of largest Vm depolarization. Accordingly, natural surround stimulation significantly reduced the likelihood that large-amplitude, depolarizing synaptic events (>3 mV change within 5 ms, see Experimental Procedures) triggered a spike in mature V1 (Figure 5D; RF versus RF + natural surround, p = 1 × 10^−5^; RF + natural surround versus RF + phase-randomized surround, p = 0.01; Kruskal-Wallis test and post hoc Mann-Whitney U test), but not in immature V1 (Figure 5E; p = 0.19, Kruskal-Wallis test), even though the number of large-amplitude events did not differ between the stimulus conditions (Figures 5F and 5G; p = 0.34 and p = 0.59 for mature and immature mice, respectively; Kruskal-Wallis test). Interestingly, even in instances of action potential firing during surround stimulation, the Vm during RF + natural surround stimulation was more hyperpolarized prior to spike generation compared to RF + phase-randomized surround stimulation in mature mice (Figure S4), suggesting that the relative magnitude of excitation and inhibition governs spike generation during full-field stimulation. Taken together, natural surround stimuli most effectively recruit precisely timed hyperpolarization to increase the selectivity of spiking to stimuli in the RF.

### Dark-Rearing Prevents the Emergence of Sensitivity for Natural Surround Stimuli

**Figure 6:**
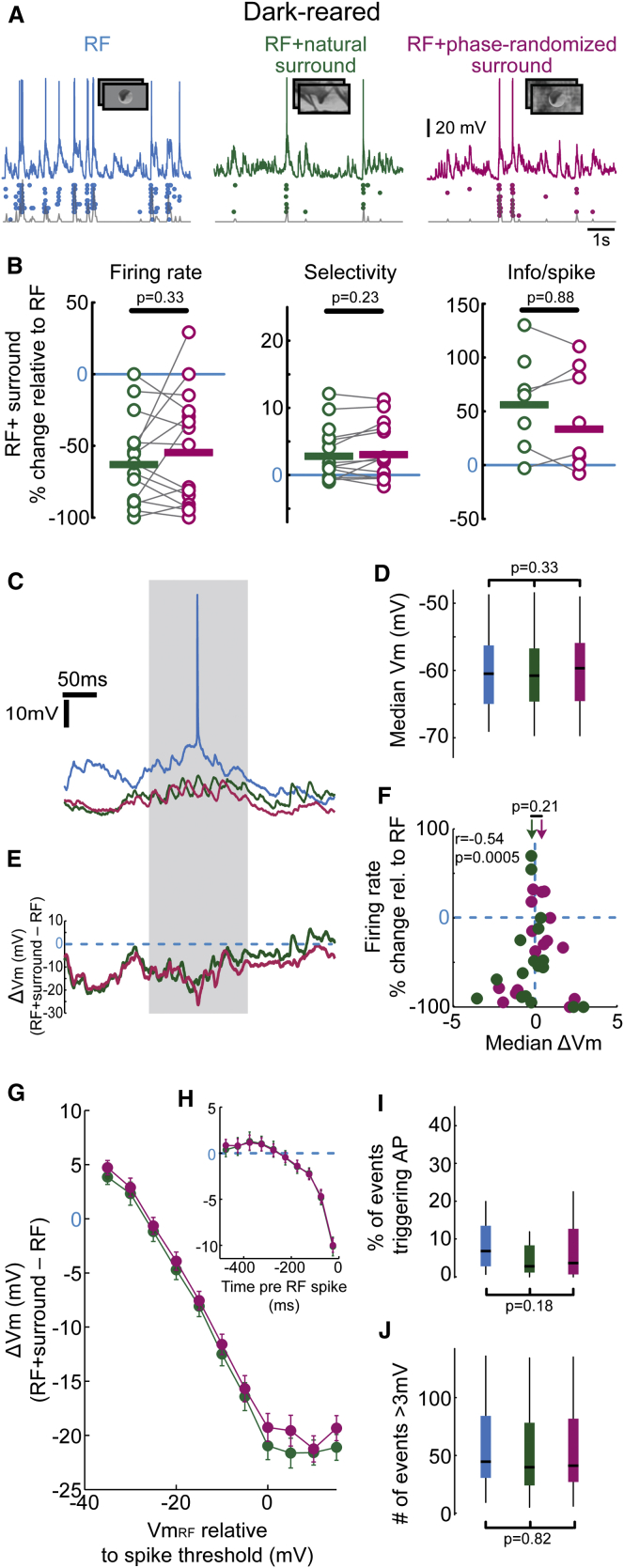
Dark-Rearing Prevents the Emergence of Preference for Natural Surround Stimuli (A) Responses from an example neuron in dark-reared, mature V1 during stimulation of the RF (blue trace), RF + natural surround (green trace), and RF + phase-randomized surround (magenta trace). (B) Analysis of spiking responses of neurons recorded in dark-reared mice. Same conventions as in Figures 2A and 2B. (C–F) Analysis of subthreshold responses of V1 neurons recorded in dark-reared mice. Same conventions as in Figures 3A–3D. (D) The median membrane depolarisation of V1 neurons in dark-reared mice (n = 19) during stimulation of RF (blue), RF + natural surround (green), or RF + phase-randomized surround (magenta) was not significantly different (RF, −60.5 mV; RF + natural surround, −60.8 mV; RF + phase-randomized surround, –59.7 mV; p = 0.33; Friedman’s test). (F) ΔVm during RF + natural surround (green) and during RF + phase-randomized surround (magenta) stimulation was not significantly different (medians indicated by arrows, p = 0.21, Wilcoxon sign-rank-test). Firing rate suppression was correlated with the median ΔVm (r = –0.54, p = 0.0005). (G) ΔVm as a function of the mean Vm depolarization during RF stimulation (bin size 5 mV) normalized to the spike threshold for each cell. ΔVm during RF + natural surround (green) and during RF + phase-randomized surround (magenta) stimulation were similar. (H) ΔVm as a function of the time before the firing of a spike during RF stimulation in dark-reared mice. ΔVm during RF + natural (green) and RF + phase-randomized (magenta) surround conditions were highly similar. (I) In dark-reared, mature V1, the likelihood for large-amplitude events to trigger a spike was not significantly different across conditions (p = 0.18, Kruskal-Wallis test across all three conditions). (J) In dark-reared, mature V1, large-amplitude, depolarizing synaptic events were similarly frequent across stimulation conditions (p = 0.82; Kruskal-Wallis test across all three conditions).

The results thus far suggest that there is an age-dependent increase in sensitivity of visual circuits for features in natural movies extending beyond the RF, which confers greater response selectivity to neurons in V1. To determine whether this increased sensitivity for the statistical structure of full-field natural scenes depends on visual experience during development, we carried out recordings in mature mice that were reared in the dark until P32–P40 and therefore never experienced normal visual input. The estimated RF size did not differ significantly between the dark-reared, immature, and normal mature mice (p = 0.65, one-way ANOVA; Figure S5). Similar to immature and mature animals, costimulation of the surround suppressed neuronal responses and increased their selectivity in dark-reared mice (Figures 6A and 6B; firing rate change RF + natural surround, –60.7% ± 7.9%, p < 0.001; RF + phase-randomized surround, –52.3% ± 10.3%, p < 0.001, n = 15; t test), indicating that the capacity of visual circuits for surround modulation was maintained and not disrupted by rearing animals devoid of visual experience. Importantly, however, we observed no significant differences between the effects of the natural and phase-randomized surrounds on responses to stimuli in the RF (Figure 6B) in terms of firing rate (RF + natural versus RF + phase-randomized surround, p = 0.33, paired t test), response selectivity (p = 0.23, paired t test), or information transmitted per spike (p = 0.88, paired t test). Differences in the level of spike suppression were not related to differences in absolute firing rates in any age group (Figure S6).

The indifference of dark-reared V1 neurons to the statistical properties of surround stimuli was also reflected at the level of subthreshold membrane potential dynamics (note that the cellular sensitivity for spiking to membrane potential changes was comparable to the other age groups; Figure S5; Azouz and Gray 2003). The median Vm in dark-reared mice was not significantly altered by costimulation of RF and surround (Figures 6C and 6D, n = 19; p = 0.33; Friedman’s test). Similar to normally reared, mature mice, there was a strong dependence of ΔVm (Figure 6E) on the level of Vm depolarization during stimulation of the RF alone (Figures 6G and 6H). However, the distribution of ΔVm was not different between natural and phase-randomized surround stimulation conditions (Figure 6F, p = 0.21, Wilcoxon rank sum test), and ΔVm at most depolarized Vm during RF stimulation was not significantly different when costimulating the surround with natural and phase-randomized stimuli (Figures 6G and 6H) in dark-reared V1, similar to the observations in immature V1 (Figures 3F–3J). Accordingly, while the likelihood of spiking during large-amplitude depolarizing events (which were unaltered in frequency of occurrence across conditions; Figure 6J; p = 0.82, Kruskal-Wallis test) was reduced, it was not significantly different between the two surround conditions (Figure 6I; p = 0.18, Kruskal-Wallis test across all conditions). These findings are consistent with a similar level of firing rate suppression by phase-randomized and natural surround stimuli (Figure 6B) in these visually inexperienced but mature animals. Thus, the emergence of neuronal sensitivity for image features extending beyond the RF boundaries requires visual experience after eye opening.

## Discussion

Our findings provide evidence for a progressive developmental refinement of visual processing to the global statistics of the natural environment, as hypothesized previously (Olshausen and Field, 1996, Berkes et al., 2011, Sachdev et al., 2012). In mouse V1 we observed developmental improvements in coding efficiency for natural scenes after eye opening (increased response selectivity and mutual information rate), which was brought about by an increased neuronal sensitivity for natural scene statistics in the RF surround, but not for surround stimuli lacking the statistical regularities of natural scenes. This emergence of efficient processing of natural stimuli was dependent on sensory experience, because it was absent in animals reared without visual input.

In cat and monkey V1, costimulation of RF and its surround with naturalistic stimuli leads to more sparse and efficient responses than during stimulation of the RF alone (Vinje and Gallant, 2000, Haider et al., 2010). Similarly, we found that in mature mouse V1, the full-field naturalistic movie was most effective for reducing spike rate and increasing selectivity and information per spike, consistent with the idea that neural codes are constrained by the same factors across mammalian species (i.e., energy consumption and information transmitted). Our findings reveal the existence of circuit mechanisms for improving coding efficiency beyond that provided by the filter characteristics of the RF alone (Olshausen and Field, 1996, David et al., 2004, Felsen et al., 2005b), which depend on the specific structure of natural scenes spanning the RF and its surround. While phase sensitivity of the surround in general has been suggested before (Guo et al., 2005, Sachdev et al., 2012, Shen et al., 2007, Xu et al., 2005), we show that the sensitivity to the spatiotemporal stimulus correlations across RF and surround is a plausible mechanism for improving neuronal selectivity. At the population level in mouse V1, recent experiments indicate on the one hand that surround suppression is orientation tuned (Self et al., 2014) and on the other hand that the representations of natural stimuli are sparser than those of phase-scrambled stimuli (Froudarakis et al., 2014). Our data not only suggest a circuit mechanism for this increased coding efficiency of natural scenes but also reveal its developmental dependency.

Importantly, while surround suppression was apparent albeit weaker already in the first days after eye opening, the surround-induced increase in response selectivity and information per spike were unspecific to the statistical properties of the surround stimuli in these visually inexperienced mice. The circuit mechanisms for increasing response selectivity are therefore present but not yet sensitive to detect the higher-order stimulus correlations of natural scenes in the immature visual pathway. Moreover, neurons in dark-reared, mature V1 were also indifferent to the statistics of surround stimuli. Visual experience, therefore, may be required to promote the refinement of neuronal circuits to detect congruent information across the field of view, which leads to improved response selectivity of mature V1 neurons for features embedded in full-field natural scenes. We note, however, that this refinement may not only depend on visual experience, as dark-rearing may also delay the development of visual circuits (Fagiolini et al., 1994, Iwai et al., 2003, Espinosa and Stryker, 2012).

Cortical inhibition likely plays a role in surround-induced response suppression in V1 (Haider et al., 2010, Adesnik et al., 2012, Nienborg et al., 2013). Our results extend this idea by revealing how costimulation of the RF surround affects membrane potential dynamics to suppress neuronal firing; while the average membrane potential was altered little by surround stimulation, the principal effect of the surround was to counteract membrane depolarization generated by stimulation of the RF alone. Specifically, we observed an experience-dependent increase of relative membrane hyperpolarization by natural surround stimuli at times of large depolarizing events during RF stimulation. This hyperpolarization was partly mediated by an increased Cl^−^ conductance, most likely through GABA_A_ receptors. Yet the average firing rates of PV and SOM interneurons, although slightly reduced by surround stimulation, were not different between natural compared to phase-randomized surround stimulation in mature V1. Hence, the preferential sensitivity for natural scene statistics in the surround was not mediated by a relative increase of inhibitory tone. Rather, we identified transient increases in membrane hyperpolarization during natural relative to phase-randomized surround stimulation, particularly at times that coincided with moments of greatest depolarization during RF stimulation. These temporal differences in the magnitude of hyperpolarization resulted in increased spike suppression, and thereby increased the response selectivity for features in full-field natural scenes in mature V1, but not in the immature or visually deprived V1. Therefore, our results suggest that sensory experience during maturation exerts a prominent influence on the recruitment of inhibition—particularly with respect to its timing relative to potential firing events—to generate more selective coding of visual features embedded in natural scenes.

Our results are broadly consistent with observations in cat V1, where there is a transient increase of inhibition during surround suppression with drifting grating stimuli (Ozeki et al., 2009), which ultimately results in an overall reduction of both excitatory and inhibitory conductances when the circuit reaches a balanced state. Our results, however, underscore the importance of transient hyperpolarization prior to spiking events as a mechanism for effective surround suppression during ongoing stimulation with natural movies. A probable explanation for this difference is that the statistical properties of grating stimuli are much narrower than that of the naturalistic stimuli used in our study. The continuous variation of spectrotemporal content of naturalistic movies may prevent cortical circuits ever reaching a stable state of balanced excitation and inhibition that is observed when using narrowband grating stimuli.

These results suggest a possible functional role for the elaboration of both excitatory and inhibitory intracortical circuits, which are susceptible to changes in sensory experience in the period after eye opening (Ruthazer and Stryker, 1996, Zufferey et al., 1999, White et al., 2001, Chattopadhyaya et al., 2004, Katagiri et al., 2007, Ko et al., 2013). We propose that circuit connectivity is shaped by exposure to the statistical structure of the natural environment (e.g., extended contours or edges) after the onset of vision, which increases the effectiveness of surround modulation when viewing naturalistic stimuli to which animals are typically exposed. Our data suggest that visual experience optimizes spiking output by refining the timing and magnitude of inhibition recruited by the surround.

In conclusion, our results support the idea that visual circuits mature in an experience-dependent manner to become sensitive to the statistical structure of natural stimuli extending beyond the boundaries of the RF. While the basic RF properties are established by the time of eye opening (Hubel and Wiesel, 1963, Blakemore and Van Sluyters, 1975, Chapman and Stryker, 1993, Krug et al., 2001, White et al., 2001, Rochefort et al., 2011, Ko et al., 2013), efficient representations of natural stimulus features—in terms of selectivity, information transfer, and energy consumption (Barlow, 1961, Simoncelli and Olshausen, 2001, Laughlin, 2001)—are not inherent to sensory circuits but require visual experience to develop.

## Experimental Procedures

### Animals and Surgery

All experimental procedures were licensed and performed in accordance with institutional and national animal welfare guidelines. Data were obtained from C57BL/6 mice aged postnatal day (P) 14–19 (immature age group, n = 7) or P32–P40 (mature age group n = 10; dark-reared age group n = 8). For dark rearing, mice were kept in complete darkness from P13 until placed under anesthesia. Mice were initially anaesthetized with a mixture of fentanyl (0.05 mg/ml), midazolam (5.0 mg/kg), and medetomidin (0.5 mg/kg). Anesthesia was maintained with a low concentration of isoflurane (typically 0.5% mixed with O2) delivered by a small nose cone. Details of the surgery are given in Supplemental Experimental Procedures.

### Receptive Field Mapping

The position and size of a neuron’s RF were determined in similar way as described before (Jones et al., 2001, Jones et al., 2002). First, RF center position was mapped with pseudorandomized sparse noise stimulus sequence (white and black flashing patches on an isoluminant gray background). Then, the RF radius was estimated by determining a circular area of half-maximal spike responses to the same pseudorandomized sparse noise stimulus. Next, we presented the naturalistic movie within an aperture centered on the RF, surrounded by an isoluminant gray (see Supplemental Experimental Procedures). These apertured naturalistic movies were interleaved with a naturalistic movie shown in the surrounding annulus of the same size (i.e., center not stimulated). We explicitly used the naturalistic movie in this procedure (rather than a grating stimulus) to achieve best estimates, because RF radius and surround effects are dependent on contrast, which is constantly fluctuating in movies, but not in grating stimuli. The radius of the aperture and the surrounding annulus were systematically varied (typically 0.4 to 2 times the originally estimated RF radius in 5 steps). This sequence was repeated at least 5 times. The aperture size that elicited the strongest response (firing rate), but no significant response to the annulus stimulus of the corresponding size, was defined as the RF size (Jones et al., 2001, Ozeki et al., 2009) (see Figure 1B). Mean and distribution of RF sizes obtained in this way were very similar for the three experimental groups (Figure S5).

Next, the naturalistic movie was presented in one of the following ways: In the RF condition, the naturalistic movie was presented within a RF-sized aperture, masking all portions of the movie outside the calculated RF with an isoluminant gray screen. To ensure a smooth transition to the surround, linear alpha-blending (0.3/°) was applied at the border of the RF and gray surround. In the natural surround condition, the naturalistic movie was shown full-field. In the phase-randomized surround condition, the natural movie was shown in the central aperture, while the phase-randomized movie covered all the surrounding portions of the screen. To determine the influence of the surround alone, the movies were additionally shown only in the annulus surrounding the central aperture. The duration of each stimulus condition was 7,000 ms. After each stimulus presentation, a constant gray screen was shown for 1,000 ms. Each condition was typically presented 11 times, and the first repetition was discarded from the analysis to eliminate onset-related effects.

Cells were included for further analysis if during at least one movie frame if any of the two RF + surround conditions elicited a significant response modulation (p < 0.01, randomized two-sided t test). There were no significant differences in the cortical recording depth between the age groups (range, 85−430 μm beneath cortical surface; 212 ± 15, 207 ± 20, and 198 ± 17 μm, mean ± SEM, for mature, immature, and dark-reared mice, respectively; p = 0.86; one-way ANOVA).

### Electrophysiology and Data Acquisition

Details are given in Supplemental Experimental Procedures. In short, pipettes were advanced into the cortex at 40° angle with a high positive pressure until the electrode tip was at the depth of approximately 100 μm (corresponding to superficial layer 2/3). The resistance of the pipettes was typically 6–8 MΩ, which were filled with a solution containing 110 mM potassium gluconate, 4 mM NaCl, 40 mM HEPES, 2 mM ATP-Mg, and 0.3 mM GTP-NaCl (adjusted to pH 7.2 with KOH, ∼290 mOSM). For experiments with elevated Cl^−^ reversal potential, 5 mM of potassium gluconate was replaced by 5 mM KCl in the internal solution. Recordings were obtained with a Multiclamp 700B amplifier (Axon Instruments, USA). The membrane potential was filtered at 50 Hz (Humbug) and digitized at 10 kHz (National Instruments, USA).

PV and SOM cells were targeted for whole-cell recordings in different transgenic mouse lines (PV-GFP mice, Meyer et al., 2002; GIN mice, Oliva et al., 2000; PV-Cre × lsl-tdTomato mice, Madisen et al., 2010, Hofer et al., 2011), using either 30 μM Alexa Fluor 594 or Alexa Fluor 488 (Life Technologies, UK) in the internal solution. The targeted cells and patch pipettes were visualized using a custom-built two-photon microscope in the green and red channels with excitation at 880 and 930 nm, respectively.

### Data Analysis

All analysis was performed with built-in or custom-made functions in Matlab (MathsWorks, USA). Selectivity index (SI) and mutual information (MI) were calculated as described before (Haider et al., 2010, Borst and Theunissen, 1999) and are explained in detail in the Supplemental Experimental Procedures.

Moment-to-moment differences in Vm (ΔVm) between RF and RF + surround conditions for each neuron were calculated in frame-wide bins (33 ms, Figures 3D and 3I) or 1 ms bins (Figures 3E and 3J) from spike-removed traces (spikes removed at spike threshold, see below). The mean ΔVm during either surround stimulation for each frame was plotted either against the mean Vm relative to spiking threshold (5 mV binning) or against the relative time before firing a spike (−500 ms to −1 ms in 50 ms bins) during the RF stimulation. Spike threshold was determined as in Haider et al. (2010). The membrane potential preceding a spike was first identified, and the membrane potential value at which the second derivative of the membrane potential was maximal was defined as threshold.

Analysis of depolarizing events was carried out by quantifying the number and size of transient positive membrane deflections. Events were detected with a moving window (bin width 5 ms) with an amplitude threshold of 3 mV. An individual event was regarded to have triggered a spike if the peak amplitude of the event was followed by an action potential.

Statistical significance for repeated measurements of the same cell with different stimuli was assessed using the paired Student’s t test and ANOVA for reaped measurements (parametric data) or Wilcoxon sign-rank test and Friedman’s test (nonparametric data).

## Author Contributions

M.P. and T.D.M.-F. conceived of the experiments and wrote the paper. E.S., Y.H., and M.P. collected while M.P. and Y.H. analyzed the data.

## Supplemental Information

Document S1. Figures S1–S6 and Supplemental Experimental Procedures

Document S2. Article plus Supplemental Information
