# Supplementary material for: Experience-Dependent Specialization of Receptive Field Surround for Selective Coding of Natural Scenes
Source: Neuron. 2014 Oct 22;84(2):457–69. doi: 10.1016/j.neuron.2014.09.010 (PMC4210638; doi:10.1016/j.neuron.2014.09.010)
Supplement: Document S1. Figures S1–S6 and Supplemental Experimental Procedures [file mmc1.pdf]

Neuron, Volume *84*

Supplemental Information

Experience-Dependent Specialization of Receptive Field Surround for Selective Coding of Natural Scenes

Michael Pecka, Yunyun Han, Elie Sader, and Thomas D. Mrsic-Flogel

Supplemental Information

Experience-dependent specialization of receptive field surround for selective coding of natural scenes

Michael Pecka, Yunyun Han, Elie Sader, and Thomas D. Mrsic-Flogel

Supplemental Inventory

Six supplemental figures with legends and supplemental Experimental Procedures:

Figure S1 relates to figure 1
Figure S2 relates to figure 2
Figure S3 relates to figures 3 and 4
Figure S4 relates to figure 5
Figure S5 relates to figures 3, 5 and 6
Figure S6 relates to figures 2 and 6

Supplemental Experimental Procedures:
Description of procedures and analyses used for data presented in figures 1 – 6 and S1 – S6.

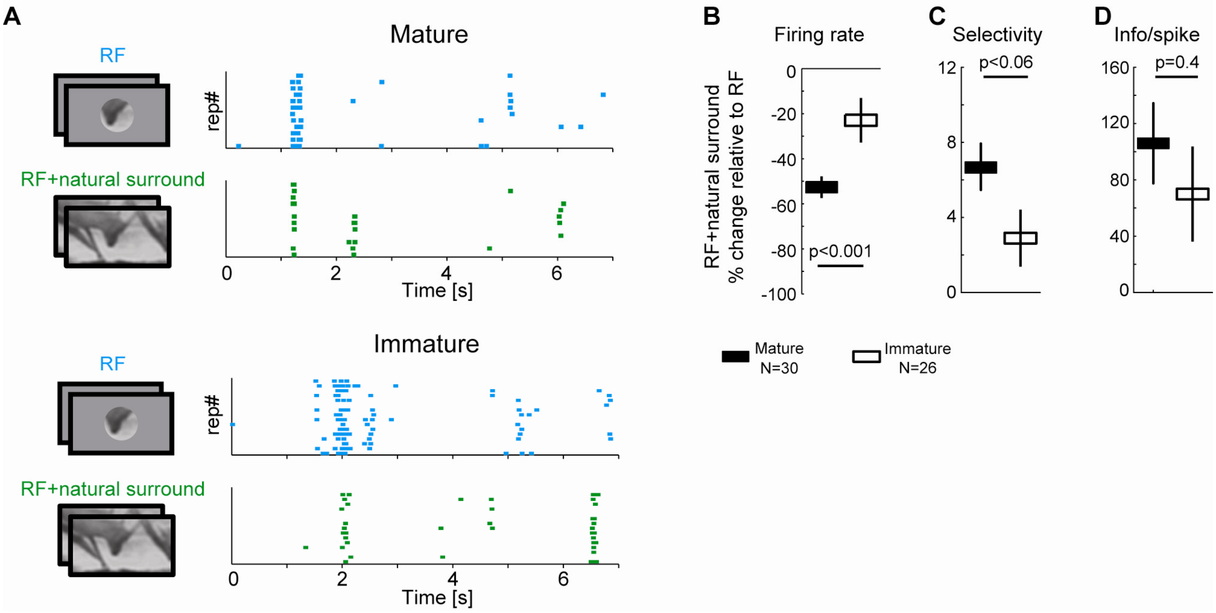

Figure S1. Surround modulation in mature and immature mice

(A) Raster plot display of an example cell from a mature (upper two panels) and immature mouse (lower two panels) recorded in juxta-cellular mode. Similar to the results from whole-cell recordings, co-stimulation of the surround with natural stimuli lead to response suppression in both age-groups.

(B)-(D) Quantifications of changes in firing rate (B), selectivity (C) and info/spike (D) for all cells recorded in juxta-cellular mode. Similar to the results from whole-cell recordings, significant decrease in firing rate and increased selectivity and information transmitted per spike were found for co-stimulation of the surround in either age-group, yet changes were stronger in mature animals than in immature animals. Conventions are as in Figure 1.

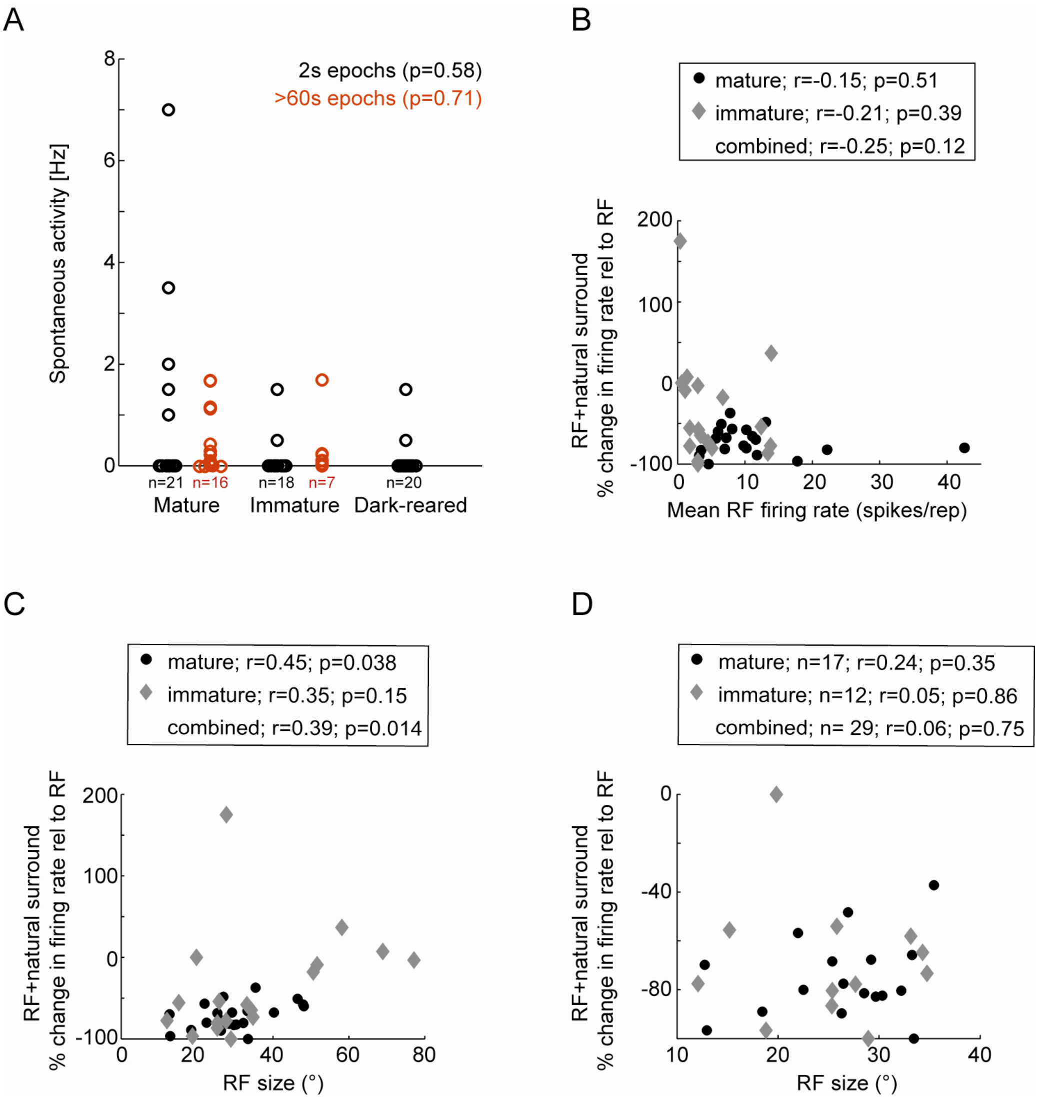

Figure S2. Surround suppression is not affected by levels of spontaneous and evoked activity or RF size.

(A) Spontaneous activity in the population of neurons (black) was measured using 2s long epochs of gray screen presentation prior to visual stimulation. Most cells exhibited negligible spontaneous rates<1Hz. Spontaneous rates were not different between age-groups (p=0.58; Friedman's test). In a separate population of cells (red), these low levels of spontaneous activity were confirmed by using long epochs (>60s) of gray screen presentation (p=0.71; Wilcoxon rank sum test).
(B) Scatter plot of firing rate suppression during RF+natural surround stimulation and the mean firing rate during RF stimulation for all neurons in mature (black filled circles) and immature (gray diamonds) mice. The level of suppression and mean RF firing rate were not correlated (Spearson's correlation and corresponding p-values are given in box above panel).
(C)-(D) Scatter plot of firing rate suppression during RF+natural surround stimulation and the RF size. Conventions as in (B). The level of suppression was mildly correlated with the RF size for data from mature mice and when combining immature and mature data. However, the correlation was predominantly caused by a few neurons woth very large RF sizes, as no correlations was found for neurons with RF sizes <40° (D, number of neurons given in box above panel).

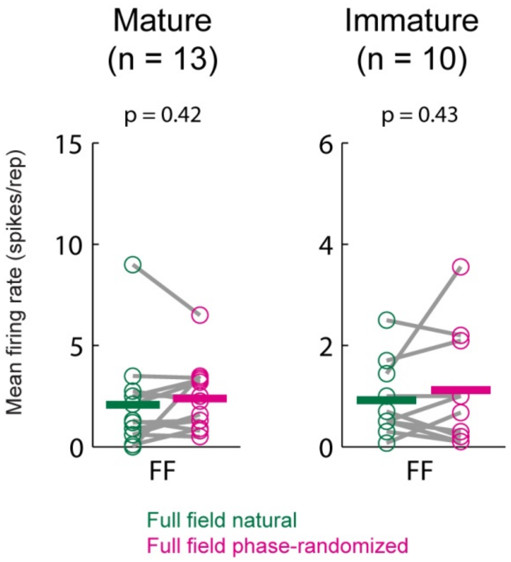

Figure S3. Full field presentation of phase-randomized movies evokes similar activity levels as natural movies.

Firing rates of Pyramidal neurons in mature (left) and immature (right) V1 were not significantly different between full field presentations of natural and phase-randomized movies. P-values derived from paired t-tests.

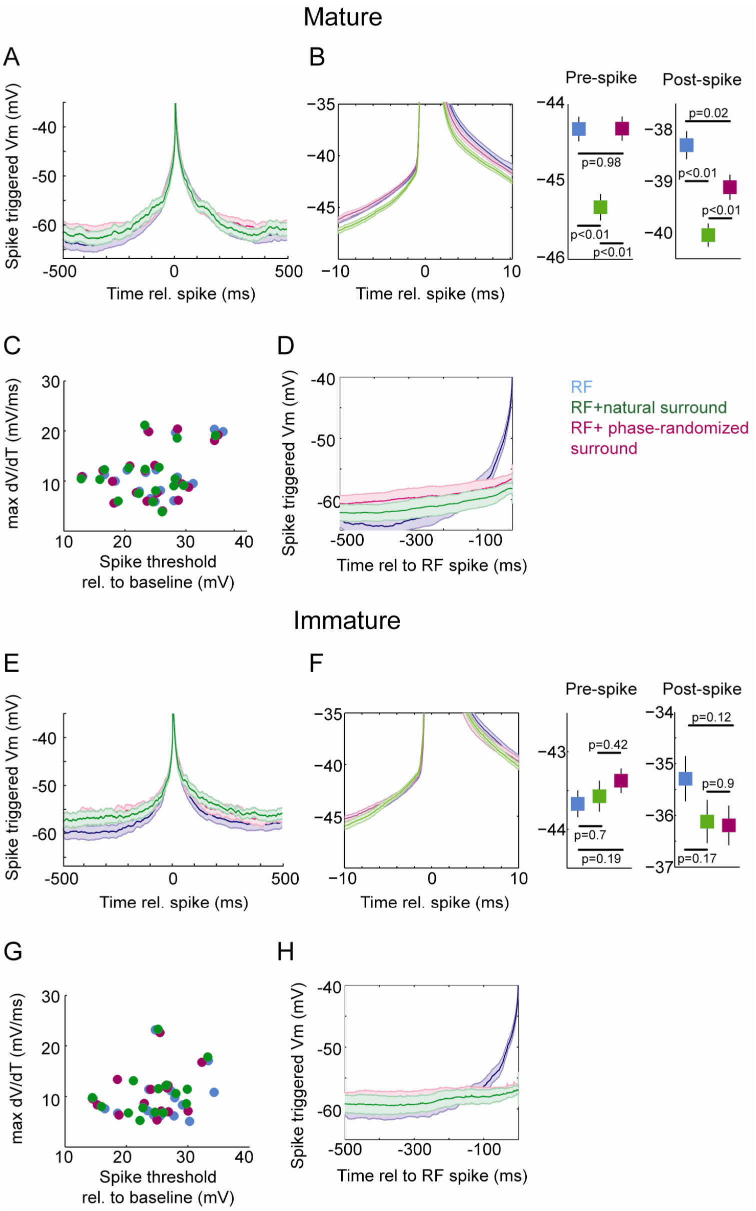

Figure S4. Average Vm prior to spiking reveals increased hyperpolarization during natural surround stimulation in mature V1.

(A) Spike-triggered averages (STAs) for all three stimulus conditions ±500ms depiciting the mean Vm (±S.E.M.) of neurons from mature mice (n=21) relative to the occurrence of a spike (see Supplemental Methods for details).
(B) Left-hand panel shows zoom-in from A) at ±10ms. RF+natural surround was significantly more hyperpolarized in the last 10ms before spiking (quantifications are shown in right-hand panels, p-values derived from t-tests)
(C) Spike threshold and maximal dV/dt did not differ across stimulus conditions.
(D) RF-STAs for the three stimulus conditions (see Supplemental Methods for details) reveal a stronger hyperpolarization during RF+natural surround compared to RF+phase-randomized surround stimulation.
(E)-(H) Same as in (A)-(D) for immature mice (n=18). In contrast to mature mice, no significant differences in hyperpolarization before spiking were present.

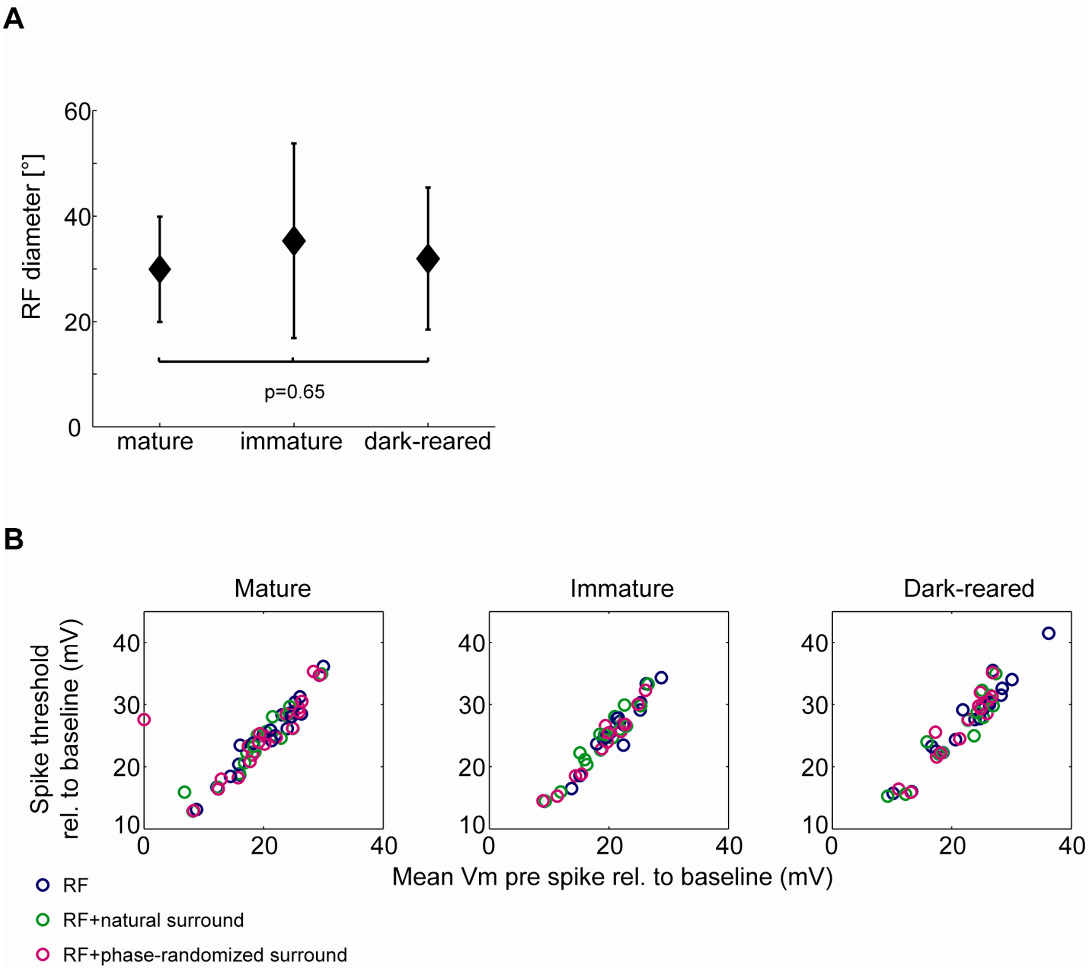

Figure S5. RF sizes and membrane properties were similar for all age-groups

(A) Mean RF diameter (+-S.E.M.) were similar in the three age-groups tested (n=21, 18, 19 for mature, immature and dark-reared, respectively; P=0.65, one-way ANOVA).
(B) Spike threshold of individual cells (open circles) is plotted as a function of the mean Vm within 10ms preceding the spike. The relationship was highly consistent both across stimulus condition and age-group.

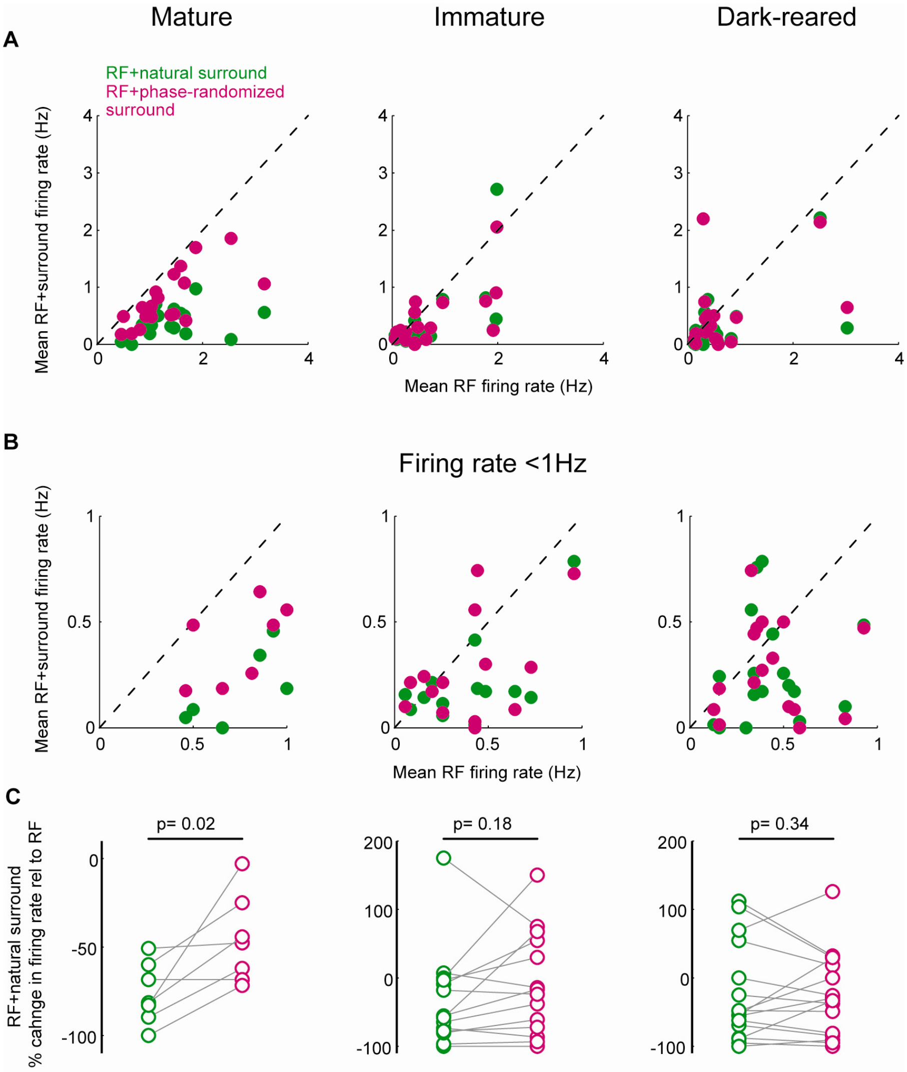

Figure S6. The level of firing rate suppression is not related to absolute firing rates.

(A) Scatter plot displays of mean RF firing rates and mean RF+surround firing rates for all neurons in the three age groups. Clearly, response suppression is most prominent in mature mice, particularly for natural surround stimulation.
(B) Scatter plots as in A) for the sub-population of cells with firing rates < 1Hz, demonstrating that the more suppressive effect of RF+surround stimulation in mature mice compared to the other age groups is not related to the overall lower firing rates in immature and dark-reared mice.
(C) Pair-wise analysis of neurons with firing rates < 1Hz confirms that absolute firing rates do not affect the sensitivity of surround suppression for natural stimuli. P-values are derived from paired t-tests.

Supplemental Experimental Procedures:

Surgery. To prevent dehydration of the cornea, a thin layer of cream (Isoptomax) was applied to the eyes before it was carefully removed prior to the start of the recordings. Animals were placed on a heating pad to maintain constant body temperature. Atropine (0.01 ml at 0.1 mg/ml) and Fortecortin (0.01 ml at 2 mg/ml) were injected subcutaneously. The skull was immobilized by affixing it to a metal plate with dental cement. A small (~1mm) craniotomy was performed above the monocular region of the visual cortex, as determined by stereotactic coordinates, and later confirmed with retinotopic mapping. A durectomy was occasionally performed to improve the quality of the recordings. Dehydration of the exposed cortical surface was prevented by regular administration of cortex buffer (150 mM NaCl, 2.5 mM KCl, 10 mM HEPES, pH 7.4). The eye ipsilateral to the craniotomy was covered to prevent any binocular stimulation.

Stimuli. Stimuli were presented to the contralateral eye on a 30'' TFT monitor (Samsung SyncMaster 305T) positioned at 27cm distance to the animal's head. The monitor was centered on the RF of the recorded neuron if possible. The frame rate was set to 30 Hz and the monitor refresh rate was set to 60 Hz. A naturalistic movie was presented consisting of multiple sequences of continuous naturalistic scenes (each typically ~1000ms in duration) with cross-fading of individual sequences (sequences were taken from David Attenborough's Life of Mammals, BBC). The resolution of the movie was set to 100 x 56 pixels (horizontal x vertical) and spatial frequencies of the movie were low-pass filtered at 0.16 cycles/degree to account for the spatial acuity of mice (Niell and Stryker, 2008), thereby maximizing the contrast of the movie within the spectrum that mice are able to detect. The mean contrast of the naturalistic movie and phase-randomized noise movies over all frames was set to 0.7. Phase-randomized movies were generated by randomizing the phases of the spatial frequency spectrum of the natural movie. Phase-randomized movies were subsequently low-pass filtered at 0.16 cycles/degree and the mean luminance and mean contrast was set to match the original naturalistic movie.

Electrophysiology and data acquisition. Pipettes were advanced into the cortex at 40o angle with a high positive pressure until the electrode tip was at the depth of approximately 100 µm (corresponding to superficial layer 2/3). The positive pressure was then reduced to 20-40 mmHg and the pipette was advanced in steps of 2.5 µm until the pipette resistance increased suddenly and a bouncy baseline detected – indicating contact with a cell. At this point the pressure was released, and slight suction was subsequently applied until a gigaseal was formed, and then the whole-cell configuration was established by applying quick steps of negative pressure. Capacitance compensation and bridge balance were monitored and carefully adjusted after obtaining the gigaseal. Except for brief current pulses to assess firing pattern, no current was injected during then recording and the membrane potential was not corrected for liquid junction potentials.

Data analysis. We calculated the lifetime response selectivity index (SI) as previously described (Vinje and Gallant, 2000; Haider et al., 2010):

$SI=\{1-[(\sum ri/n)2/\sum(ri2/n)]\}/[1-(1/n)]$,

where ri is the average response to the ith frame of the movie, and n is the number of movie frames. The resulting values of SI are bounded between 0 and 1. A value of 1 indicates responses to only one frame in all repetitions, while a value of 0 indicates completely

random or unselective firing to any frame. Importantly, SI has a sigmoid shape as a function of frame selectivity with asymptotic behavior near 0 and 1. In our dataset, SI was generally rather high (typically > 0.8), hence significant changes in selectivity resulted in only modest relative changes in SI.

Information transmission was measured by using a direct method to calculate the average mutual information (Shannon, 1948) as: MI= H(R) - H(R|S), where H(R) is the spiking response entropy and H(R|S) is the noise entropy. Spike trains were divided into 15 ms time windows to obtain binary response trains on which noise and response entropies were calculated as described previously (Borst and Theunissen, 1999; Magri et al., 2009). Time windows of 5 ms, 33 ms (movie frame duration) and 60 ms were also tested and yielded qualitatively similar results. To compensate for any overestimation of MI caused by the limited number of trails, we applied the analytical Panzeri-Treves bias correction (Panzeri and Treves, 1996; Magri et al., 2009). A shuffling bias correction procedure was additionally performed (Panzeri et al 2007), but did not further influence the results. To determine the significance of the calculated information for each neuron, 100 repetitions of MI calculations with randomized binary response trains were performed using bootstrapping methods (Magri et al., 2009). A neuron was only considered for further analysis if MI of the original spike train exceeded the mean MI +2*S.D. of the randomized spike trains. Values of MI were divided by the respective number of spikes to give the information for each spike (information/spike) (Vinje and Gallant, 2002). For comparisons of firing rate, SI and mutual information/spike across age-groups (Fig. 2), analysis was restricted to cells that exhibited significant firing rate suppression in response to at least one surround condition.

Spike-triggered averages (STA) were computed by averaging the membrane potential recordings for ±500ms relative to the peak voltage deflection of a spike. To eliminate confounding influences on the mean Vm, voltage traces were only included in this analysis if spikes were not preceded or followed by another spike within +-500ms. For RF-STAs, time periods were determined based on the occurrences of spikes during the RF condition only. The Vm during RF+surround stimulation were analyzed for the equivalent time periods as in the RF condition regardless whether spikes had occurred in the RF+surround condition.

Supplemental References:

Magri, C., Whittingstall, K., Singh, V., Logothetis, N.K., and Panzeri, S. (2009). A toolbox for the fast information analysis of multiple-site LFP, EEG and spike train recordings. BMC Neurosci 10, 81.

Panzeri, S., and Treves, A. (1996). Analytical estimates of limited sampling biases in different information measures. Network: Computation in Neural Systems 7, 87–107.

Panzeri, S., Senatore, R., Montemurro, M.A., and Petersen, R.S. (2007). Correcting for the Sampling Bias Problem in Spike Train Information Measures. J Neurophysiol 98, 1064–1072.

Shannon, C.J. (1948). A mathematical theory of communication. Bell Systems Techincal Journal 27, 379–423.
