# Supplementary material for: Experience-Dependent Specialization of Receptive Field Surround for Selective Coding of Natural Scenes
Source: Neuron. 2014 Oct 22;84(2):457–69. doi: 10.1016/j.neuron.2014.09.010 (PMC4210638; doi:10.1016/j.neuron.2014.09.010)
Supplement: Document S2. Article plus Supplemental Information [file mmc2.pdf]

Neuron
Article

Experience-Dependent Specialization of Receptive Field Surround for Selective Coding of Natural Scenes

Michael Pecka,[1,3,4,*] Yunyun Han,[1,2,3] Elie Sader,[1,3] and Thomas D. Mrsic-Flogel[1,2,*]
[1]Department of Neuroscience, Physiology, and Pharmacology, University College London, 21 University Street, London WC1E 6DE, UK
[2]Biozentrum, University of Basel, Klingelbergstrasse 50/70, 4056 Basel, Switzerland
[3]Co-first author
[4]Present address: Division of Neurobiology, Department II, Biocenter of the Ludwig-Maximilian-University Munich, Grosshaderner Strasse 2, 82152 Planegg-Martinsried, Germany
*Correspondence: pecka@bio.lmu.de (M.P.), thomas.mrsic-flogel@unibas.ch (T.D.M.-F.)
http://dx.doi.org/10.1016/j.neuron.2014.09.010
This is an open access article under the CC BY license (http://creativecommons.org/licenses/by/3.0/).

SUMMARY

At eye opening, neurons in primary visual cortex (V1) are selective for stimulus features, but circuits continue to refine in an experience-dependent manner for some weeks thereafter. How these changes contribute to the coding of visual features embedded in complex natural scenes remains unknown. Here we show that normal visual experience after eye opening is required for V1 neurons to develop a sensitivity for the statistical structure of natural stimuli extending beyond the boundaries of their receptive fields (RFs), which leads to improvements in coding efficiency for full-field natural scenes (increased selectivity and information rate). These improvements are mediated by an experience-dependent increase in the effectiveness of natural surround stimuli to hyperpolarize the membrane potential specifically during RF-stimulus epochs triggering action potentials. We suggest that neural circuits underlying surround modulation are shaped by the statistical structure of visual input, which leads to more selective coding of features in natural scenes.

INTRODUCTION

The visual system is specialized to extract features from complex natural scenes that have a unique statistical structure (Simoncelli and Olshausen, 2001; Felsen et al., 2005a), including edges and contours that change in space and time across the field of view. Although neurons in the primary visual cortex (V1) respond best to local image features that fall within their receptive fields (RFs), their responses are strongly modulated by stimuli placed in the surrounding regions of visual space (Blakemore and Tobin, 1972; Nelson and Frost, 1978; Allman et al., 1985; Gilbert and Wiesel, 1990). Typically, stimulating the surround suppresses responses to stimuli in the RF (Jones et al., 2001, 2002; Seriès et al., 2003; Ozeki et al., 2009; Adesnik et al., 2012), and this suppression is more pronounced when using natural surround images than when using their phase-scrambled versions devoid of complex structure (Guo et al., 2005). Visual circuits are thus particularly sensitive to integrating salient image components across natural scenes, which may contribute to contour integration and "pop-out" phenomena at the perceptual level (Knierim and van Essen, 1992). Concomitantly, surround modulation by natural images alters the firing rate distribution of individual neurons, whereby their responses become more selective and sparse (Vinje and Gallant, 2000, 2002; Haider et al., 2010). Sparse codes are considered efficient, because they are able to transfer more information with fewer spikes (Olshausen and Field, 2004). Taken together, surround modulation contributes to contextual processing of sensory information and increases the efficiency of neural representations for natural scenes (Sachdev et al., 2012).

How do neural circuits become specialized to integrate and efficiently represent information from complex natural scenes, which contain image features that extend beyond the RF of any individual neuron? Neurons in V1 are feature selective already at eye opening (Hubel and Wiesel, 1963; Blakemore and Van Sluyters, 1975; Chapman and Stryker, 1993; Krug et al., 2001; White et al., 2001; Rochefort et al., 2011; Ko et al., 2013). However, little is known about the development of surround modulation and its dependence on early sensory experience, and how this impacts the ability to encode complex natural scenes. Surround modulation is mediated by excitatory and inhibitory interactions at different stages of the mature visual pathway, including the retina (Olveczky et al., 2003; Solomon et al., 2006) and visual cortex (Stettler et al., 2002; Angelucci and Bressloff, 2006; Girardin and Martin, 2009; Ozeki et al., 2009; Haider et al., 2010; Adesnik et al., 2012; Nienborg et al., 2013; Vaiceliunaite et al., 2013). Since both excitatory and inhibitory circuits refine after eye opening (Frégnac and Imbert, 1984; Katagiri et al., 2007; Kuhlman et al., 2011; Ko et al., 2013) and are susceptible to changes in visual experience (Ruthazer and Stryker, 1996; Zufferey et al., 1999; White et al., 2001; Chattopadhyaya et al., 2004; Maffei et al., 2004), the effectiveness of surround modulation may be expected to change during postnatal development. The extent to which this may improve the processing of full field natural scenes is, however, unknown.

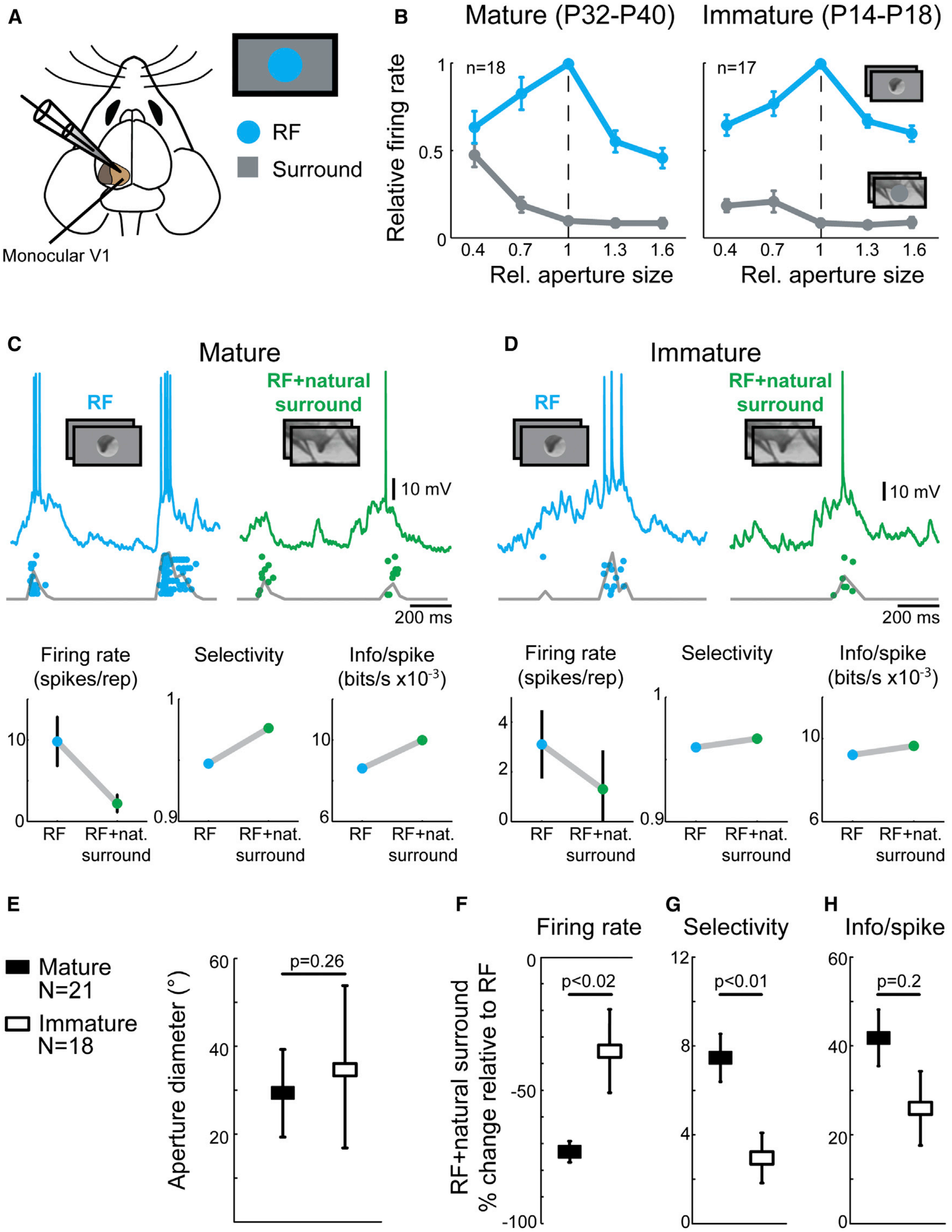

Figure 1. Surround-Induced Increases in Response Suppression and Selectivity Are Present in Immature V1 but Are More Pronounced in Mature V1

(A) Schematic depicting whole-cell patch-clamp recordings of neurons in the monocular region of primary visual cortex (V1) of anesthetized mice. The size of the receptive field (RF) and the influence of the surround were determined by presentation of a naturalistic movie to the contralateral eye, by varying the size of the center (aperture) and surrounding (annulus) stimuli.

(B) Averaged normalized size-tuning functions of V1 neurons in mature and immature mice. The mean normalized firing rates (±SEM) at aperture sizes relative to optimal RF size ("1") are shown for apertured (RF only) and corresponding annulus (surround only) stimuli. Only neurons with similar increments in aperture size were included for this analysis (mature, n = 18; immature, n = 17).

(legend continued on next page)

In this study, we show that circuits mediating surround modulation require sensory experience to become preferentially sensitive to natural stimulus statistics across the RF and its surround. In mature mouse V1, neuronal firing to natural movies presented to the RF became more selective when costimulating the surround with natural movies than with phase-scrambled movies lacking the higher-order statistical regularities of natural scenes. In contrast, this preferential sensitivity of center-surround interactions for natural scenes was absent in immature, visually naive V1 after eye opening and in mature animals that were reared without visual input. Mechanistically, the surround-induced increase of response selectivity was mediated by transient membrane potential hyperpolarization that coincided with moments of greatest depolarization during RF stimulation. These transient hyperpolarizing events were most effective in limiting spiking during full-field natural movie stimulation in adult V1, consistent with the increased effectiveness of the natural surround stimuli in improving response selectivity. Therefore, normal visual experience is required for the refinement of neuronal circuits that contribute to the selective coding of natural scenes by spatially integrating information from the entire field of view.

RESULTS

Surround Suppression in Mature and Developing Mouse V1

To study the effectiveness of surround modulation during postnatal development, we carried out in vivo whole-cell recordings from individual neurons in cortical layer 2/3 of monocular V1 in immature mice with limited visual experience (1–5 days after eye opening, P14–P19, n = 18 from 7 mice) and in visually mature mice with at least 18 days of normal visual experience (P32–P40, n = 21 from 10 mice). To determine the exact RF size of each recorded neuron, we alternated the presentation of a naturalistic movie within apertures of increasing size (isoluminant gray surround) and the corresponding surround (annulus) regions (Figure 1A; see Experimental Procedures). In both mature and immature V1, neuronal firing was stimulus size dependent (Figure 1B). Responses first increased and then decreased with increasing aperture size, while response rate decreased for the corresponding surround stimuli (Figure 1B, see figure legend for details). The RF size—defined by the aperture diameter at which neurons exhibited a maximal response without a significant response to the corresponding annulus stimulus—was similar for the two age groups (Figure 1E; mean ± SEM, mature, 29.9° ± 10°; immature, 35.3° ± 18°, p = 0.26, t test). While responses decreased significantly during full-field stimulation with natural movies (RF + natural surround; Figures 1C and 1D) compared to stimulation of the RF alone (p < 0.01 for both mature and immature mice, paired t test), they were suppressed more in mature V1 (Figure 1F, mature, −71.9% ± 3.6%; immature, −35.3% ± 15.6%, p = 0.019, t test). These results show that neurons in immature V1 exhibit surround suppression within a few days after eye opening, but that the suppressive effect of the surround becomes stronger with age.

To determine how the developmental strengthening of surround suppression influences the selectivity of neuronal responses, we computed a measure that captures the distribution of spiking across stimulus repetitions (here referred to as "selectivity," also known as "lifetime sparseness"; Vinje and Gallant, 2002; Lehky et al., 2005; Tolhurst et al., 2009; Willmore et al., 2011; see Experimental Procedures). In both immature and mature V1, response selectivity increased significantly during natural surround stimulation compared to stimulation of the RF alone (Figure 1G; p < 0.01, paired t test), and this increase was significantly greater in mature animals (mature, 7.5% ± 1.1%; immature, 3.0% ± 1.1%, p = 0.008, t test).

A reduced spike rate and increased selectivity only add to the efficiency of a neuronal representation if the information about the stimulus is adequately maintained (Laughlin, 2001; Vinje and Gallant, 2002). Hence, the amount of information per spike should increase to compensate for fewer evoked spikes. In both age groups, costimulating the surround significantly increased the information per spike (see Experimental Procedures) relative to the stimulation confined to the RF (Figure 1H, p < 0.01, paired t test). This increase tended to be higher in mature than in immature V1 (mature, 41.9% ± 6.3%; immature, 26.2% ± 8.2%, p = 0.2, t test), but the effect did not reach significance. Very similar results were obtained in a separate data set using juxtacellular single-cell recordings (Figure S1 available online), indicating that any alterations of the intracellular milieu caused by the whole-cell recording technique did not influence the results. These age-dependent effects of the surround on firing rate suppression were not influenced by any differences in RF size or absolute firing rate between of neurons recorded in the two age groups (Figure S2). Taken together, these data indicate that visual circuits are capable of spatial integration already at eye opening, but that surround modulation becomes more effective at suppressing firing and increasing response selectivity to natural scenes with age.

Natural Surround Increases Response Selectivity More Than Artificial Surround in Mouse V1

In adult monkey V1, the effectiveness of surround modulation depends on the higher-order structure of natural scenes (e.g., extended contours), because responses to natural images in the RF are suppressed less when randomizing the phase of

(C) Example whole-cell recording from a neuron in mature V1 during stimulation of RF or RF + natural surround. Voltage traces of a single repetition are shown with spike-dot-raster and spike-histogram of ten repetitions overlaid underneath the trace. Lower panels show metrics derived from this example recording, including mean firing rate (±SEM, left panel), selectivity index (middle panel), and mutual information/spike (right panel).
(D) Example whole-cell recording from a neuron in immature V1 (postnatal day 18) during stimulation of the RF and RF + natural surround. Conventions as in (C).
(E) Mean aperture (RF) diameter (±SEM) for all neurons recorded in mature (29.9° ± 10°; n = 21) and immature mice (35.3° ± 18°, n = 18) was not significantly different (p = 0.26, t test).
(F–H) Mean population changes (%± SEM) in firing rate (F), selectivity (G), and mutual information/spike (H) during RF + natural surround stimulation normalized to RF stimulation for mature (black symbols) and immature (white symbols) mice. p values refer to differences in mean change across age groups (t test).

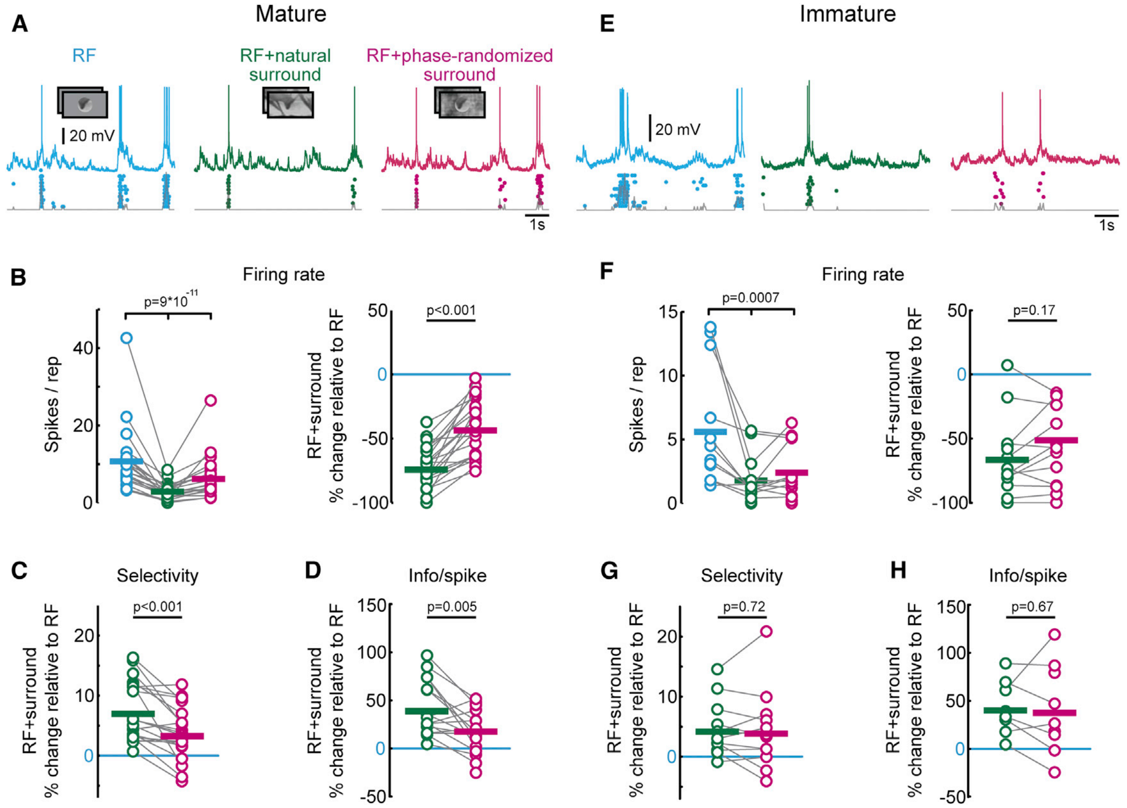

Figure 2. Neurons Are Sensitive to Natural Stimulus Statistics in the RF Surround in Mature, but Not in Immature, V1

(A) Example whole-cell recording from a layer 2/3 neuron in V1 of a mature mouse (postnatal day 36) during stimulation of the RF (left panel, blue trace), RF + natural surround (middle panel, green trace), and RF + phase-randomized surround (right panel, magenta trace). Conventions as in Figure 1C.

(B) Firing rates during the three stimulus conditions (left panel), and changes (%) in firing rate (right panel) during stimulation of the RF + natural surround (green) or RF + phase-randomized surround (magenta) relative to stimulating the RF center in mature mice. Mean firing rates for each cell are shown by connected open circles. Horizontal bars denote group mean values.

(C and D) Changes (%) in selectivity (C) and information/spike (D) during stimulation of the RF + natural surround (green) or RF + phase-randomized surround (magenta) relative to stimulating the RF center in mature mice (n = 21 cells; p values, paired t test).

(E) Example whole-cell recording from a L2/3 neuron in immature V1 (postnatal day 16). Conventions as in (A).

(F) Firing rate during the three stimulus conditions (left panel), and changes (%) in firing rate (right panel) during stimulation of the RF + natural surround (green) or RF + phase-randomized surround (magenta) relative to stimulating the RF center in immature mice (n = 13 cells).

(G and H) Changes (%) in selectivity (G) and information/spike (H) during stimulation of the RF + natural surround (green) or RF + phase-randomized surround (magenta) relative to stimulating the RF center in immature mice. (n = 13 cells; p values, paired t test).

natural images in the surround (Guo et al., 2005). We therefore tested whether neurons in mature mouse V1 also exhibit the dependency of RF-surround interactions on the statistical properties of surround stimuli. We compared how responses to the natural movie presented in the RF were altered by costimulating the surround either with the same natural movie (RF + natural surround) or with the phase-randomized version of the same movie (RF + phase-randomized surround, Figure 2A). Note that the phase randomization only removes the higher-order structure in natural images without altering their contrast or spatial frequency composition (see Experimental Procedures). Accordingly, full-screen presentations of natural and phase-randomized stimuli evoked similar activity levels in both age groups (Figure S3). For the following analysis, we only included neurons whose responses were significantly suppressed in at least one of the surround conditions (mature, 21/21 cells; immature, 13/18 cells; see Experimental Procedures). In mature V1, costimulation of the surround with both natural and phase-randomized stimuli reduced firing rates significantly (Figure 2B; $p = 9 \times 10^{-11}$, one-way ANOVA), increased response selectivity (Figure 2C; RF + natural surround, 7.5% ± 1.1%, $p < 0.001$; RF + phase-randomized surround, 3.7% ± 0.9%, $p < 0.001$; t test) and mutual information per spike (Figure 2D; RF + natural surround, 41.8% ± 7.4%, $p < 0.001$; RF + phase-randomized surround, 20.6% ± 6.2%, $p < 0.001$; t test) compared to stimulation of the RF alone. Importantly, however, stimulating the surround with natural movies decreased firing rates significantly more than phase-randomized surround movies (Figure 2B; $p < 0.001$, paired t test). This led to significantly greater increases in both selectivity and mutual information per spike during natural compared to phase-randomized surround stimulation (Figures 2C and 2D; $p < 0.001$ and $p = 0.005$, respectively, paired

t test). Thus, neurons in mature V1 are sensitive to the higher-order regularities of natural stimuli extending beyond the RF boundary, which makes their responses more selective and informationally efficient.

Preference for Natural Surround Stimuli Emerges during Development

We next determined whether the increased sensitivity of V1 neurons for natural surround stimuli is already apparent within a few days after eye opening. In immature mice, the costimulation of the RF with either natural or phase-randomized surround stimuli generated significant spike rate suppression (Figures 2E and 2F, $p = 0.0007$, one-way ANOVA), increased response selectivity (Figure 2G, natural surround, 4.7% ± 1.3%, $p < 0.001$; phase-randomized surround, 4.3% ± 1.8%, $p < 0.001$; t test), and information transmitted per spike (Figure 2H, natural surround, 43.2% ± 7.8%, $p < 0.001$; phase-randomized surround, 40.7% ± 12.8%, $p < 0.001$; t test). However, neither the amount of response suppression nor the increase in response selectivity and information per spike was significantly different between the two types of surround stimuli (Figures 2F–2H; $p = 0.17$, $p = 0.72$ and $p = 0.67$, respectively; paired t test). Thus, in contrast to experienced animals, neurons in immature V1 did not differentiate between naturalistic and phase-scrambled stimuli in the surround, suggesting that early circuits mediating surround modulation are not yet preferentially sensitive for higher-order structure of natural scenes extending beyond the RF.

Selective Hyperpolarization during Center-Surround Interactions Leads to Greatest Response Suppression to Full-Field Natural Scenes

We next investigated whether the age-dependent increase in the sensitivity of center-surround interactions for natural scenes can be explained by differences in subthreshold membrane potential dynamics during different stimulus conditions (Figures 3A and 3F). Previous reports indicate that surround stimulation leads to more hyperpolarized membrane potential (Vm) relative to RF stimulation alone (Haider et al., 2010, 2013), which is partly attributed to increased inhibition in the cortical network (Haider et al., 2010; Adesnik et al., 2012; Nienborg et al., 2013; Vaiceliunaite et al., 2013). However, when averaged over the entire stimulation period, we found that costimulation of the surround with either natural or phase-scrambled movies slightly depolarized the median absolute Vm in immature and mature mice (Figures 3B and 3H; $p = 0.017$ and $p < 0.0001$, respectively; Friedman's test).

Because it is unclear how such small average differences in Vm could contribute to changes in the spiking response selectivity, we focused our analysis on how Vm temporal dynamics are altered by surround stimulation. We quantified moment-to-moment differences in Vm between RF and full-field stimulation for each neuron ($\Delta Vm = Vm_{RF+surround} - Vm_{RF}$; see Experimental Procedures). Both natural and phase-randomized surround stimuli induced hyperpolarizing (negative ΔVm) and depolarizing (positive ΔVm) Vm changes relative to RF stimulation alone (Figures 3C and 3G). Plotting the median ΔVm of each cell against its average change in firing rate revealed that ΔVm was strongly correlated with the firing rate suppression during full-field stimulation in mature, but not in immature mice (Figures 3D and 3I; see figure legend for details). Moreover, the distribution of ΔVm was shifted to more negative values during natural than phase-randomized surround stimulation in mature V1 (Figure 3D, $p = 0.027$, Wilcoxon rank sum test), but not in immature V1 (Figure 3I, $p = 0.6$, Wilcoxon rank sum test).

How could relatively small differences in ΔVm between natural and phase-randomized surround stimulation lead to pronounced differences in firing rate suppression incurred by these surround stimuli in mature V1? To address this question, we determined the dependency of ΔVm on the particular membrane potential value (relative to spike threshold) elicited by the RF stimulus at each time point during movie presentation (Vm_{RF}). Strikingly, in both age groups, ΔVm exhibited a negative linear dependency on membrane depolarization during RF stimulation: neurons were relatively most hyperpolarized during RF + surround stimulation (negative ΔVm) specifically at times when Vm_{RF} was closest to spiking threshold (Figures 3E and 3J).

GABAergic Inhibition Contributes to Selective Hyperpolarization during Surround Stimulation

Which mechanisms underlie the pronounced surround-induced relative hyperpolarization when the Vm is most depolarized during RF stimulation? Surround stimulation has been shown to increase synaptic inhibition (Haider et al., 2010, 2013; Adesnik et al., 2012). We therefore tested the influence of chloride (Cl^-)-mediated conductances on the inverse relationship between ΔVm and Vm_{RF}. We performed whole-cell recordings using an elevated Cl^- concentration in the intracellular solution ($[Cl^-]_i$, see Experimental Procedures) to modify the reversal potential of $GABA_A$-mediated conductances (Figure 4A). Compared to data recorded with the normal Cl^--concentration (Figure 3E), recordings with elevated $[Cl^-]_i$ revealed a rightward shift of ΔVm values in both natural and phase-randomized surround conditions at all values of Vm_{RF} (Figure 4B). These data suggest that an increased Cl^- conductance during surround stimulation at least in part contributes to the negative relationship of ΔVm and Vm_{RF}. The Cl^- conductance may also account for the depolarizing effect of surround costimulation (positive ΔVm values, Figure 4B) at very hyperpolarized levels of Vm_{RF}, if these fall below the reversal potential for $GABA_A$-mediated conductances.

Given that the increased Cl^- conductance is most likely mediated by $GABA_A$ receptors, we explored the likely sources of GABAergic inputs by targeting parvalbumin (PV) and somatostatin (SOM) inhibitory interneurons with whole-cell recordings (Figures 4C and 4D; see Experimental Procedures). We found that firing rates of PV and SOM neurons were on average only slightly but significantly reduced by costimulation of the surround relative to stimulation of the RF alone, irrespective of the surround stimulus type (Figure 4E).The relative firing rate decrease was smaller in both PV and SOM cells compared to putative pyramidal (Pyr) cells during either surround stimulus condition (Figure 4F, RF + natural surround, Pyr-PV $p = 8 \times 10^{-6}$; Pyr-SOM $p = 8 \times 10^{-6}$; RF + phase-scrambled surround, Pyr-PV $p = 0.01$; Pyr-SOM $p = 0.01$, Mann-Whitney U test). Since PV and SOM cells maintained relatively high firing rates during RF + surround stimulation, both interneuron classes can be expected to contribute to the increased inhibition of Pyr cells during surround stimulation.

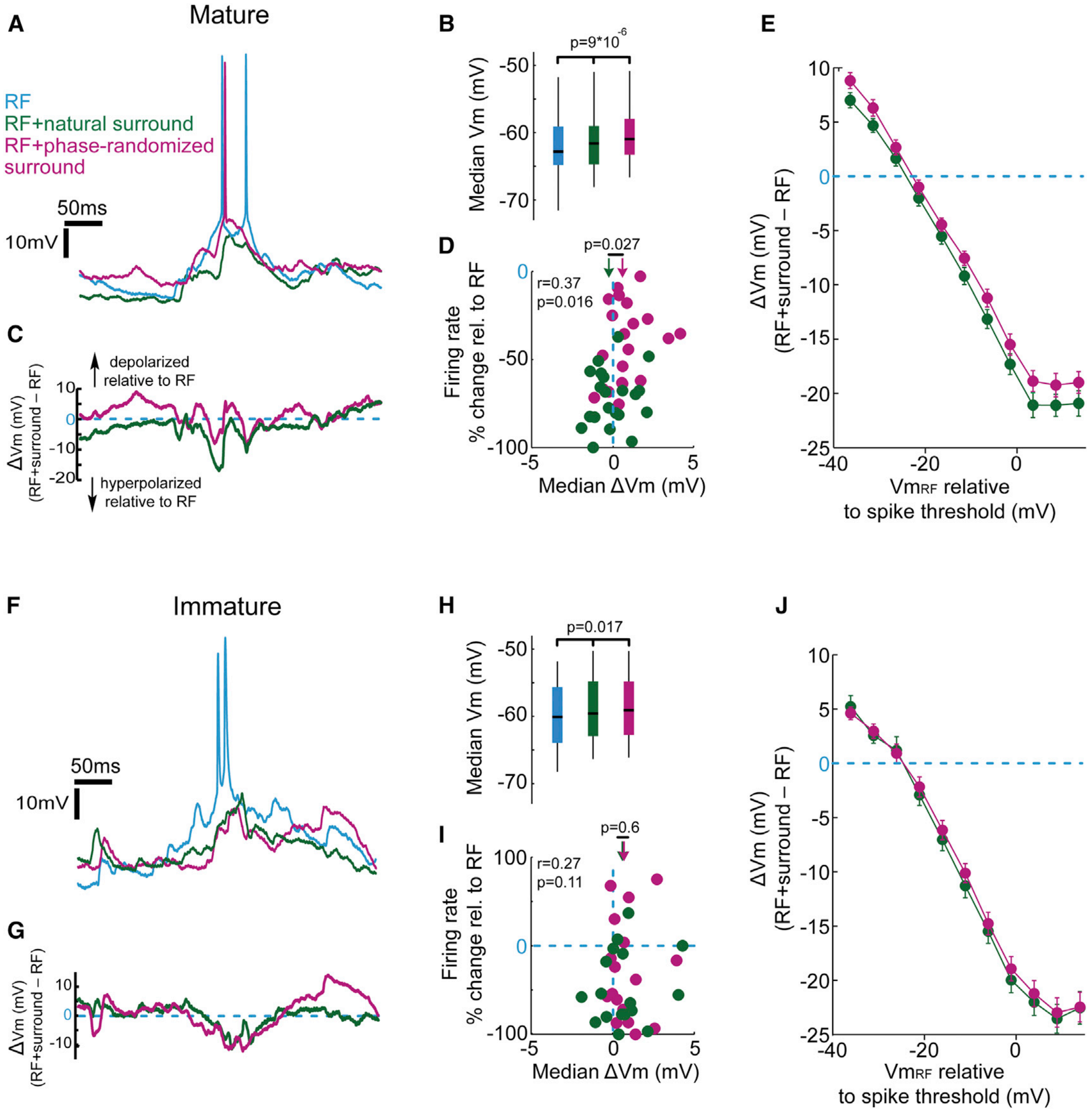

Figure 3. Natural and Phase-Randomized Surround Stimulation Elicits Significantly Different Hyperpolarization during RF Spiking Events in Mature, but Not Immature, V1

(A) Example recording from a L2/3 neuron in mature V1 (postnatal day 36) during presentation of the same movie sequence confined to the RF (blue) or when costimulating the surround with natural (green) or phase-randomized (magenta) movie.

(B) The median Vm of mature V1 neurons (n = 21) during stimulation of the RF (blue), RF + natural surround (green) and RF + phase-randomized surround (magenta) was significantly different (RF, −62.8 mV; RF + natural surround, −61.6 mV; RF + phase-randomized surround, −60.9 mV; $p = 9 \times 10^{-6}$; Friedman's test). Black mark inside colored box denote median values, while the box edges denote the 25th and 75th percentiles and the whiskers extending to the most extreme data points.

(C) ΔVm obtained by subtracting the example traces in (A) after removal of spikes.

(D) Median ΔVm during RF + natural surround stimulation (green) was significantly more negative than during RF + phase-randomized surround stimulation (magenta) (arrows denote population medians, p = 0.027, Wilcoxon sign-rank test). Firing rate suppression was strongly correlated with the median ΔVm (r = 0.37, p = 0.016).

(E) Average ΔVm (±SEM) as a function of the Vm_{RF} (bin size 5 mV). ΔVm values in each bin were averaged after normalizing to the spike threshold for each cell.

(F) Example recording from a L2/3 neuron in immature V1 (postnatal day 16) during presentation of the same movie sequence confined to the RF (blue) or when costimulating the surround with natural (green) or phase-randomized (magenta) movie.

(G) ΔVm obtained by subtracting the example traces in (F) after removal of spikes.

(H) The median Vm of immature V1 neurons (n = 18) during stimulation of the RF (blue), RF + natural surround (green) and RF + phase-randomized surround (magenta) was significantly different (RF, –60.1 mV; RF + natural surround, –59.6 mV; RF + phase-randomized surround, –59.1 mV; p = 0.017; Friedman's test).

(I) The median ΔVm during RF + natural surround stimulation (green) and during RF + phase-randomized surround stimulation (magenta) was not significantly different (population medians indicated by arrows, p = 0.6, Wilcoxon sign-rank test). Firing rate suppression was not correlated with the mean ΔVm (r = 0.27, p = 0.11).

(J) Average ΔVm as a function of the Vm_{RF} (bin size 5 mV). ΔVm values in each bin were averaged after normalizing to the spike threshold for each cell in immature mice.

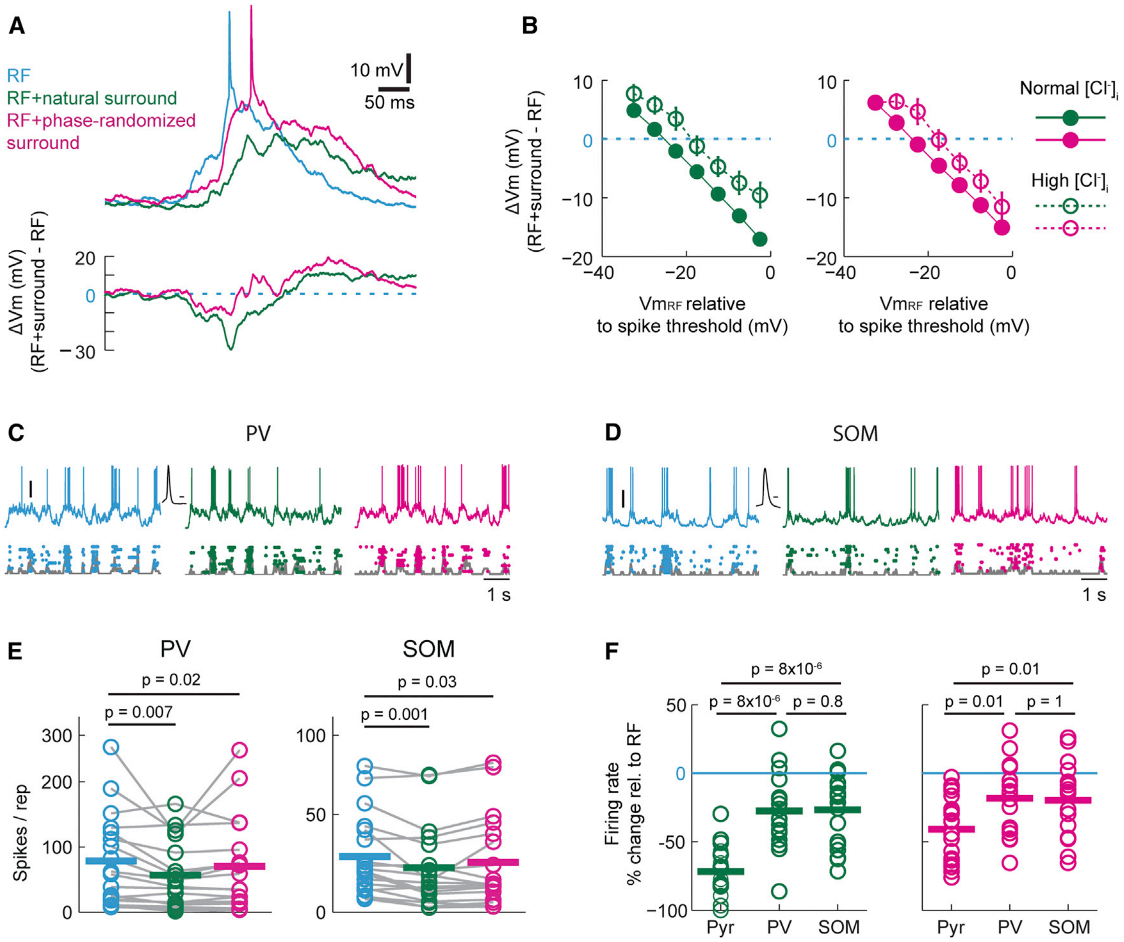

Figure 4. GABAergic Inhibition Contributes to Dependency of ΔVm on Vm_{RF}

(A) Example recording with elevated [Cl⁻] of the internal solution from a V1 neuron in a mature mouse during presentation of the same movie sequence confined to the RF (blue) or during costimulation of the surround with natural (green) or phase-randomized (magenta) movies. Lower panel shows the ΔVm for the two surround conditions.

(B) Elevated [Cl⁻] (open circles, n = 7) causes a rightward shift in the relationship between ΔVm and Vm_{RF} over the entire data range for both RF + natural (green) and RF + phase-randomized surround (magenta) conditions. See text for a detailed description. Data with normal [Cl⁻] are replotted from Figure 3E.

(C) Example targeted whole-cell recording from a parvalbumin (PV)-positive interneuron in V1 from a mature PV-GFP mouse during stimulation of the RF (left panel, blue trace), RF + natural surround (middle panel, green trace), and RF + phase-randomized surround (right panel, magenta trace). Conventions as in Figure 2A. Scale bar, 20 mV. The black trace shows the action potential waveform of the example cell. Scale bar, 1 ms.

(D) Example targeted whole-cell recording from a somatostatin (SOM) positive interneuron in V1 from a mature GIN mouse. Conventions as in (C).

(E) Firing rates of individual parvalbumin (PV, n = 18) and somatostatin (SOM, n = 18) interneurons during stimulation of the RF center (blue), RF + natural surround (green) or RF + phase-randomized surround (magenta) in mature mice. Surround stimulation resulted in slight but significant decreases in firing rates (p values of paired comparisons given in the panel, Wilcoxon signed rank test). Conventions as in Figure 2.

(F) Comparison of relative firing rates (% of firing rate during RF stimulation) of putative pyramidal cells (Pyr, n = 21), PV (n = 18), and SOM (n = 18) interneurons during costimulation of the surround with either RF + natural (green, left panel) or RF + phase-randomized (magenta, right panel) movies. PV and SOM neurons were significantly less suppressed during either surround condition compared to Pyr cells (p values of comparisons given in the panel, Mann-Whitney U test).

Importantly, neither PV nor SOM cells were preferentially selective for the natural and phase-randomized surround stimuli (Figure 4F, PV p = 0.12 and SOM p = 0.14, for RF + natural versus RF + phase-randomized surround, paired t test). This is consistent with the observation that the rightward shift of the ΔVm and Vm_{RF} relationship after elevating $[Cl^-]_i$ was not associated with a change in the slope of the relationship (Figure 4B), suggesting that surround stimuli of different statistics cause no major difference in the average increase of Cl^- conductance. These data further imply an additional involvement of other, most likely excitatory conductances, suggesting that surround suppression is rooted in the modulation of temporally balanced excitation and inhibition (Ozeki et al., 2009).

Surround-Induced Hyperpolarization at Times of Spike Generation during RF Stimulation

Our results thus far suggest that the increased response suppression and selectivity of putative pyramidal neurons during

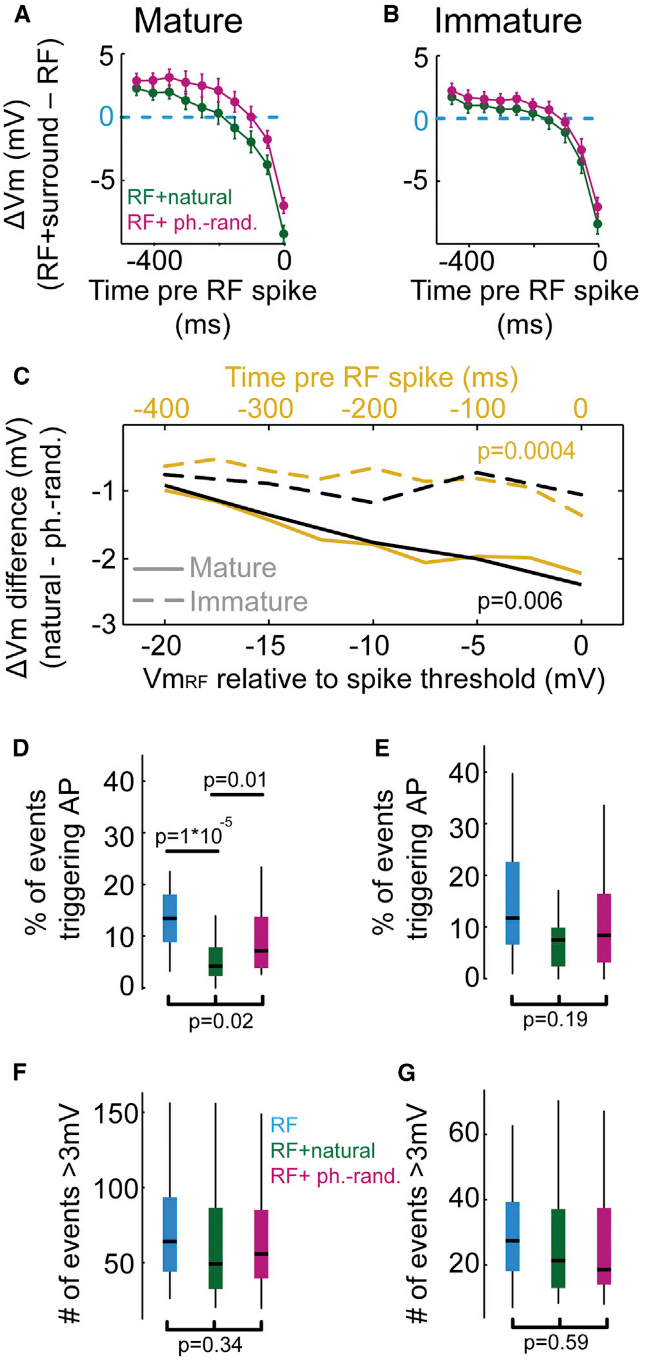

Figure 5. Precisely Timed Hyperpolarization Prior to RF Spiking Events Mediates Selective Surround Suppression

(A) ΔVm as a function of the time before a spike during RF stimulation in mature mice. The mean ΔVm (±SEM) is plotted for the corresponding times (bin = 50 ms) during RF + natural (green) and RF + phase-randomized surround (magenta) conditions. ΔVm was significantly different between the surround conditions ($F_{interaction}$ = 143, $p < 0.00001$, two-way ANOVA).

(B) ΔVm as a function of the time before a spike during RF stimulation in immature mice. ΔVm was significantly different between the surround conditions ($F_{interaction}$ = 103, $p < 0.00001$, two-way ANOVA).

(C) Quantification of differences in ΔVm between RF + natural and RF + phase-randomized surround conditions in mature (solid lines) and immature (dashed lines) mice. Differences in ΔVm between surround conditions were consistently larger for mature mice, both when analyzed relative to Vm_{RF} (black, compare Figures 3E and 3J) and relative to time of RF spike (yellow, compare A and B). Note that only in mature mice differences in ΔVm between RF + natural and RF + phase-randomized surround conditions were large at times prior RF spiking (yellow solid line, p = 0.0004, t test) and increased with increasing depolarization of Vm_{RF} (black solid line, p = 0.006, t test). Thus, these differences in ΔVm might underlie the increased firing rate suppression during RF + natural surround stimulation.

(D–G) Same conventions as in Figure 3B. (D) In mature V1, natural surround stimulation reduced the likelihood for large-amplitude synaptic events to trigger a spike in mature V1 (RF versus RF + natural surround, $p = 1 \times 10^{-5}$; RF + natural surround versus RF + phase-randomized surround, p = 0.01; Mann-Whitney U test). (E) In immature V1, the likelihood for large-amplitude synaptic events to trigger a spike was not significantly different across conditions (p = 0.19, Kruskal-Wallis test across all three conditions). (F) In mature V1, large-amplitude, depolarizing synaptic events were similarly frequent across stimulation conditions (p = 0.34; Kruskal-Wallis test across all three conditions). (G) In immature V1, large-amplitude, depolarizing synaptic events were similarly frequent across stimulation conditions (p = 0.59; Kruskal-Wallis test across all three conditions).

RF + natural surround stimulation in mature mice could not be explained by a net difference in the amount of inhibition during the two surround conditions. Instead, our results raise the possibility that the timing of inhibition may be important for this selective suppression, because the difference in ΔVm between natural and phase-randomized surround stimulation was largest at most depolarized Vm_{RF} (i.e., closest to spike threshold) in mature mice (Figure 5C; see also Figure 3E). To examine the timing of surround-induced hyperpolarization in more detail, we determined the temporal progression of ΔVm before the occurrence of a spike during RF stimulation (see Experimental Procedures). At times preceding action potential firing events during RF stimulation (corresponding to instances when the Vm is most depolarized, Figure 5C), natural surround stimuli hyperpolarized the Vm more than phase-randomized surround stimuli (Figures 5A, 5C, and S4D). This difference in the relative hyperpolarization between natural and phase-randomized surround (ΔVm difference) was significantly larger in mature mice compared to immature mice (Figures 5A–5C and S4H), both when ΔVm was binned relative to Vm_{RF} (p = 0.006, t test) and relative to RF spike time (p = 0.0004, t test). These findings are consistent with the greatest spike rate suppression during natural surround stimulation in mature V1 (Figures 2B and 3D), and suggest that suppression is caused by time-locked Vm hyperpolarization that curtails spike generation at moments of largest Vm depolarization. Accordingly, natural surround stimulation significantly reduced the likelihood that large-amplitude, depolarizing synaptic events (>3 mV change within 5 ms, see Experimental Procedures) triggered a spike in mature V1 (Figure 5D; RF versus RF + natural surround, $p = 1 \times 10^{-5}$; RF + natural surround versus RF + phase-randomized surround, p = 0.01; Kruskal-Wallis test and post hoc Mann-Whitney U test), but not in immature V1 (Figure 5E; p = 0.19, Kruskal-Wallis test), even though the number of large-amplitude events did not differ between the stimulus conditions (Figures 5F and 5G; p = 0.34 and p = 0.59 for mature and immature mice, respectively; Kruskal-Wallis test). Interestingly, even in instances of action potential firing during surround stimulation, the Vm during RF + natural surround

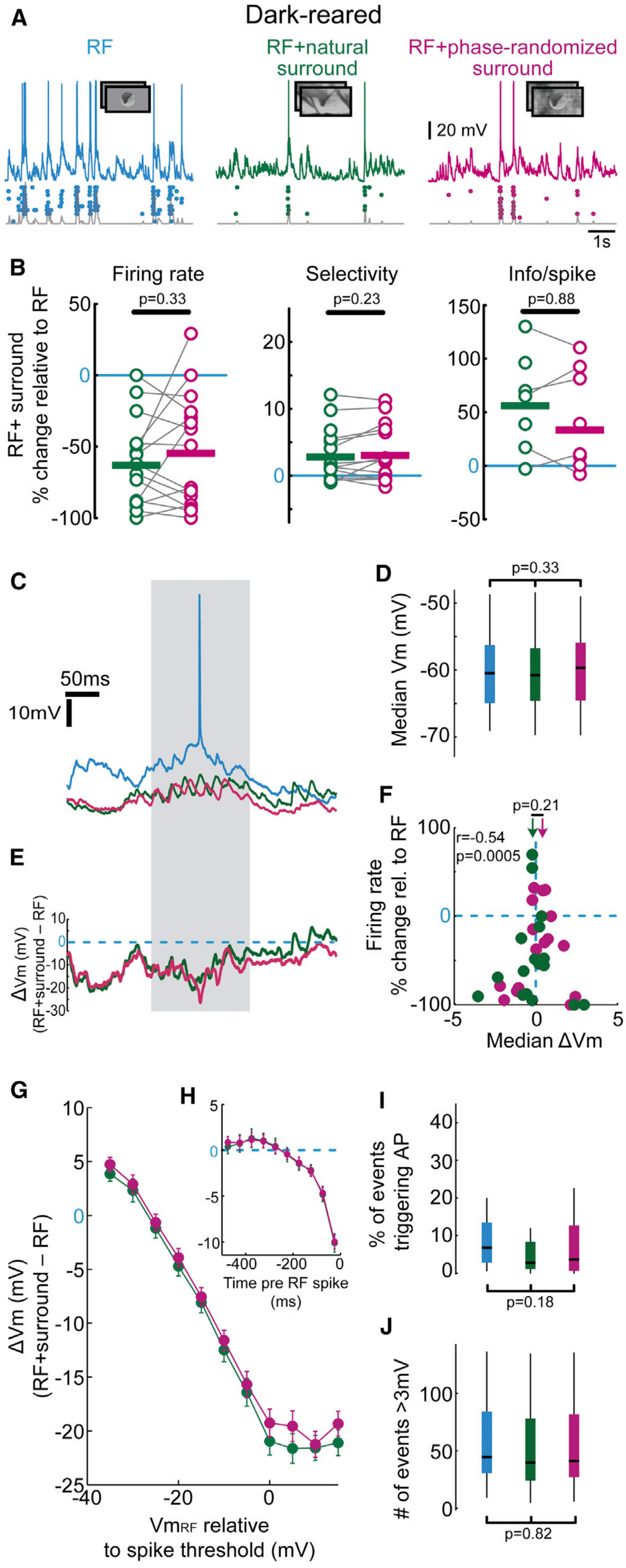

Figure 6. Dark-Rearing Prevents the Emergence of Preference for Natural Surround Stimuli

(A) Responses from an example neuron in dark-reared, mature V1 during stimulation of the RF (blue trace), RF + natural surround (green trace), and RF + phase-randomized surround (magenta trace).

(B) Analysis of spiking responses of neurons recorded in dark-reared mice. Same conventions as in Figures 2A and 2B.

(C–F) Analysis of subthreshold responses of V1 neurons recorded in dark-reared mice. Same conventions as in Figures 3A–3D.

(D) The median membrane depolarisation of V1 neurons in dark-reared mice (n = 19) during stimulation of RF (blue), RF + natural surround (green), or RF + phase-randomized surround (magenta) was not significantly different (RF, −60.5 mV; RF + natural surround, −60.8 mV; RF + phase-randomized surround, –59.7 mV; p = 0.33; Friedman's test).

(F) ΔVm during RF + natural surround (green) and during RF + phase-randomized surround (magenta) stimulation was not significantly different (medians indicated by arrows, p = 0.21, Wilcoxon sign-rank-test). Firing rate suppression was correlated with the median ΔVm (r = –0.54, p = 0.0005).

(G) ΔVm as a function of the mean Vm depolarization during RF stimulation (bin size 5 mV) normalized to the spike threshold for each cell. ΔVm during RF + natural surround (green) and during RF + phase-randomized surround (magenta) stimulation were similar.

(H) ΔVm as a function of the time before the firing of a spike during RF stimulation in dark-reared mice. ΔVm during RF + natural (green) and RF + phase-randomized (magenta) surround conditions were highly similar.

(I) In dark-reared, mature V1, the likelihood for large-amplitude events to trigger a spike was not significantly different across conditions (p = 0.18, Kruskal-Wallis test across all three conditions).

(J) In dark-reared, mature V1, large-amplitude, depolarizing synaptic events were similarly frequent across stimulation conditions (p = 0.82; Kruskal-Wallis test across all three conditions).

stimulation was more hyperpolarized prior to spike generation compared to RF + phase-randomized surround stimulation in mature mice (Figure S4), suggesting that the relative magnitude of excitation and inhibition governs spike generation during full-field stimulation. Taken together, natural surround stimuli most effectively recruit precisely timed hyperpolarization to increase the selectivity of spiking to stimuli in the RF.

Dark-Rearing Prevents the Emergence of Sensitivity for Natural Surround Stimuli

The results thus far suggest that there is an age-dependent increase in sensitivity of visual circuits for features in natural movies extending beyond the RF, which confers greater response selectivity to neurons in V1. To determine whether this increased sensitivity for the statistical structure of full-field natural scenes depends on visual experience during development, we carried out recordings in mature mice that were reared in the dark until P32–P40 and therefore never experienced normal visual input. The estimated RF size did not differ significantly between the dark-reared, immature, and normal mature mice (p = 0.65, one-way ANOVA; Figure S5). Similar to immature and mature animals, costimulation of the surround suppressed neuronal responses and increased their selectivity in dark-reared mice (Figures 6A and 6B; firing rate change RF + natural surround, –60.7% ± 7.9%, p < 0.001; RF + phase-randomized surround, –52.3% ± 10.3%, p < 0.001, n = 15; t test), indicating that the capacity of visual circuits for surround modulation was maintained and not disrupted by rearing animals devoid of visual experience. Importantly, however, we observed no significant differences between

the effects of the natural and phase-randomized surrounds on responses to stimuli in the RF (Figure 6B) in terms of firing rate (RF + natural versus RF + phase-randomized surround, $p = 0.33$, paired t test), response selectivity ($p = 0.23$, paired t test), or information transmitted per spike ($p = 0.88$, paired t test). Differences in the level of spike suppression were not related to differences in absolute firing rates in any age group (Figure S6).

The indifference of dark-reared V1 neurons to the statistical properties of surround stimuli was also reflected at the level of subthreshold membrane potential dynamics (note that the cellular sensitivity for spiking to membrane potential changes was comparable to the other age groups; Figure S5; Azouz and Gray 2003). The median Vm in dark-reared mice was not significantly altered by costimulation of RF and surround (Figures 6C and 6D, $n = 19$; $p = 0.33$; Friedman's test). Similar to normally reared, mature mice, there was a strong dependence of ΔVm (Figure 6E) on the level of Vm depolarization during stimulation of the RF alone (Figures 6G and 6H). However, the distribution of ΔVm was not different between natural and phase-randomized surround stimulation conditions (Figure 6F, $p = 0.21$, Wilcoxon rank sum test), and ΔVm at most depolarized Vm during RF stimulation was not significantly different when costimulating the surround with natural and phase-randomized stimuli (Figures 6G and 6H) in dark-reared V1, similar to the observations in immature V1 (Figures 3F–3J). Accordingly, while the likelihood of spiking during large-amplitude depolarizing events (which were unaltered in frequency of occurrence across conditions; Figure 6J; $p = 0.82$, Kruskal-Wallis test) was reduced, it was not significantly different between the two surround conditions (Figure 6I; $p = 0.18$, Kruskal-Wallis test across all conditions). These findings are consistent with a similar level of firing rate suppression by phase-randomized and natural surround stimuli (Figure 6B) in these visually inexperienced but mature animals. Thus, the emergence of neuronal sensitivity for image features extending beyond the RF boundaries requires visual experience after eye opening.

DISCUSSION

Our findings provide evidence for a progressive developmental refinement of visual processing to the global statistics of the natural environment, as hypothesized previously (Olshausen and Field, 1996; Berkes et al., 2011; Sachdev et al., 2012). In mouse V1 we observed developmental improvements in coding efficiency for natural scenes after eye opening (increased response selectivity and mutual information rate), which was brought about by an increased neuronal sensitivity for natural scene statistics in the RF surround, but not for surround stimuli lacking the statistical regularities of natural scenes. This emergence of efficient processing of natural stimuli was dependent on sensory experience, because it was absent in animals reared without visual input.

In cat and monkey V1, costimulation of RF and its surround with naturalistic stimuli leads to more sparse and efficient responses than during stimulation of the RF alone (Vinje and Gallant, 2000; Haider et al., 2010). Similarly, we found that in mature mouse V1, the full-field naturalistic movie was most effective for reducing spike rate and increasing selectivity and information per spike, consistent with the idea that neural codes are constrained by the same factors across mammalian species (i.e., energy consumption and information transmitted). Our findings reveal the existence of circuit mechanisms for improving coding efficiency beyond that provided by the filter characteristics of the RF alone (Olshausen and Field, 1996; David et al., 2004; Felsen et al., 2005b), which depend on the specific structure of natural scenes spanning the RF and its surround. While phase sensitivity of the surround in general has been suggested before (Guo et al., 2005; Sachdev et al., 2012; Shen et al., 2007; Xu et al., 2005), we show that the sensitivity to the spatiotemporal stimulus correlations across RF and surround is a plausible mechanism for improving neuronal selectivity. At the population level in mouse V1, recent experiments indicate on the one hand that surround suppression is orientation tuned (Self et al., 2014) and on the other hand that the representations of natural stimuli are sparser than those of phase-scrambled stimuli (Froudarakis et al., 2014). Our data not only suggest a circuit mechanism for this increased coding efficiency of natural scenes but also reveal its developmental dependency.

Importantly, while surround suppression was apparent albeit weaker already in the first days after eye opening, the surround-induced increase in response selectivity and information per spike were unspecific to the statistical properties of the surround stimuli in these visually inexperienced mice. The circuit mechanisms for increasing response selectivity are therefore present but not yet sensitive to detect the higher-order stimulus correlations of natural scenes in the immature visual pathway. Moreover, neurons in dark-reared, mature V1 were also indifferent to the statistics of surround stimuli. Visual experience, therefore, may be required to promote the refinement of neuronal circuits to detect congruent information across the field of view, which leads to improved response selectivity of mature V1 neurons for features embedded in full-field natural scenes. We note, however, that this refinement may not only depend on visual experience, as dark-rearing may also delay the development of visual circuits (Fagiolini et al., 1994; Iwai et al., 2003; Espinosa and Stryker, 2012).

Cortical inhibition likely plays a role in surround-induced response suppression in V1 (Haider et al., 2010; Adesnik et al., 2012, Nienborg et al., 2013). Our results extend this idea by revealing how costimulation of the RF surround affects membrane potential dynamics to suppress neuronal firing; while the average membrane potential was altered little by surround stimulation, the principal effect of the surround was to counteract membrane depolarization generated by stimulation of the RF alone. Specifically, we observed an experience-dependent increase of relative membrane hyperpolarization by natural surround stimuli at times of large depolarizing events during RF stimulation. This hyperpolarization was partly mediated by an increased Cl^{-} conductance, most likely through $GABA_A$ receptors. Yet the average firing rates of PV and SOM interneurons, although slightly reduced by surround stimulation, were not different between natural compared to phase-randomized surround stimulation in mature V1. Hence, the preferential sensitivity for natural scene statistics in the surround was not mediated by a relative increase of inhibitory tone. Rather, we identified transient increases in membrane hyperpolarization

during natural relative to phase-randomized surround stimulation, particularly at times that coincided with moments of greatest depolarization during RF stimulation. These temporal differences in the magnitude of hyperpolarization resulted in increased spike suppression, and thereby increased the response selectivity for features in full-field natural scenes in mature V1, but not in the immature or visually deprived V1. Therefore, our results suggest that sensory experience during maturation exerts a prominent influence on the recruitment of inhibition—particularly with respect to its timing relative to potential firing events—to generate more selective coding of visual features embedded in natural scenes.

Our results are broadly consistent with observations in cat V1, where there is a transient increase of inhibition during surround suppression with drifting grating stimuli (Ozeki et al., 2009), which ultimately results in an overall reduction of both excitatory and inhibitory conductances when the circuit reaches a balanced state. Our results, however, underscore the importance of transient hyperpolarization prior to spiking events as a mechanism for effective surround suppression during ongoing stimulation with natural movies. A probable explanation for this difference is that the statistical properties of grating stimuli are much narrower than that of the naturalistic stimuli used in our study. The continuous variation of spectrotemporal content of naturalistic movies may prevent cortical circuits ever reaching a stable state of balanced excitation and inhibition that is observed when using narrowband grating stimuli.

These results suggest a possible functional role for the elaboration of both excitatory and inhibitory intracortical circuits, which are susceptible to changes in sensory experience in the period after eye opening (Ruthazer and Stryker, 1996; Zufferey et al., 1999; White et al., 2001; Chattopadhyaya et al., 2004; Katagiri et al., 2007; Ko et al., 2013). We propose that circuit connectivity is shaped by exposure to the statistical structure of the natural environment (e.g., extended contours or edges) after the onset of vision, which increases the effectiveness of surround modulation when viewing naturalistic stimuli to which animals are typically exposed. Our data suggest that visual experience optimizes spiking output by refining the timing and magnitude of inhibition recruited by the surround.

In conclusion, our results support the idea that visual circuits mature in an experience-dependent manner to become sensitive to the statistical structure of natural stimuli extending beyond the boundaries of the RF. While the basic RF properties are established by the time of eye opening (Hubel and Wiesel, 1963; Blakemore and Van Sluyters 1975; Chapman and Stryker, 1993; Krug et al., 2001; White et al., 2001; Rochefort et al., 2011; Ko et al., 2013), efficient representations of natural stimulus features—in terms of selectivity, information transfer, and energy consumption (Barlow, 1961; Simoncelli and Olshausen, 2001; Laughlin, 2001)—are not inherent to sensory circuits but require visual experience to develop.

EXPERIMENTAL PROCEDURES

Animals and Surgery

All experimental procedures were licensed and performed in accordance with institutional and national animal welfare guidelines. Data were obtained from C57BL/6 mice aged postnatal day (P) 14–19 (immature age group, n = 7) or P32–P40 (mature age group n = 10; dark-reared age group n = 8). For dark rearing, mice were kept in complete darkness from P13 until placed under anesthesia. Mice were initially anaesthetized with a mixture of fentanyl (0.05 mg/ml), midazolam (5.0 mg/kg), and medetomidin (0.5 mg/kg). Anesthesia was maintained with a low concentration of isoflurane (typically 0.5% mixed with O2) delivered by a small nose cone. Details of the surgery are given in Supplemental Experimental Procedures.

Receptive Field Mapping

The position and size of a neuron's RF were determined in similar way as described before (Jones et al., 2001, 2002). First, RF center position was mapped with pseudorandomized sparse noise stimulus sequence (white and black flashing patches on an isoluminant gray background). Then, the RF radius was estimated by determining a circular area of half-maximal spike responses to the same pseudorandomized sparse noise stimulus. Next, we presented the naturalistic movie within an aperture centered on the RF, surrounded by an isoluminant gray (see Supplemental Experimental Procedures). These apertured naturalistic movies were interleaved with a naturalistic movie shown in the surrounding annulus of the same size (i.e., center not stimulated). We explicitly used the naturalistic movie in this procedure (rather than a grating stimulus) to achieve best estimates, because RF radius and surround effects are dependent on contrast, which is constantly fluctuating in movies, but not in grating stimuli. The radius of the aperture and the surrounding annulus were systematically varied (typically 0.4 to 2 times the originally estimated RF radius in 5 steps). This sequence was repeated at least 5 times. The aperture size that elicited the strongest response (firing rate), but no significant response to the annulus stimulus of the corresponding size, was defined as the RF size (Jones et al., 2001; Ozeki et al., 2009) (see Figure 1B). Mean and distribution of RF sizes obtained in this way were very similar for the three experimental groups (Figure S5).

Next, the naturalistic movie was presented in one of the following ways: In the RF condition, the naturalistic movie was presented within a RF-sized aperture, masking all portions of the movie outside the calculated RF with an isoluminant gray screen. To ensure a smooth transition to the surround, linear alpha-blending (0.3/°) was applied at the border of the RF and gray surround. In the natural surround condition, the naturalistic movie was shown full-field. In the phase-randomized surround condition, the natural movie was shown in the central aperture, while the phase-randomized movie covered all the surrounding portions of the screen. To determine the influence of the surround alone, the movies were additionally shown only in the annulus surrounding the central aperture. The duration of each stimulus condition was 7,000 ms. After each stimulus presentation, a constant gray screen was shown for 1,000 ms. Each condition was typically presented 11 times, and the first repetition was discarded from the analysis to eliminate onset-related effects.

Cells were included for further analysis if during at least one movie frame if any of the two RF + surround conditions elicited a significant response modulation ($p < 0.01$, randomized two-sided t test). There were no significant differences in the cortical recording depth between the age groups (range, 85−430 μm beneath cortical surface; 212 ± 15, 207 ± 20, and 198 ± 17 μm, mean ± SEM, for mature, immature, and dark-reared mice, respectively; p = 0.86; one-way ANOVA).

Electrophysiology and Data Acquisition

Details are given in Supplemental Experimental Procedures. In short, pipettes were advanced into the cortex at 40° angle with a high positive pressure until the electrode tip was at the depth of approximately 100 μm (corresponding to superficial layer 2/3). The resistance of the pipettes was typically 6–8 MΩ, which were filled with a solution containing 110 mM potassium gluconate, 4 mM NaCl, 40 mM HEPES, 2 mM ATP-Mg, and 0.3 mM GTP-NaCl (adjusted to pH 7.2 with KOH, ~290 mOSM). For experiments with elevated Cl^- reversal potential, 5 mM of potassium gluconate was replaced by 5 mM KCl in the internal solution. Recordings were obtained with a Multiclamp 700B amplifier (Axon Instruments, USA). The membrane potential was filtered at 50 Hz (Humbug) and digitized at 10 kHz (National Instruments, USA).

PV and SOM cells were targeted for whole-cell recordings in different transgenic mouse lines (PV-GFP mice, Meyer et al., 2002; GIN mice, Oliva et al.,

2000; PV-Cre × lsl-tdTomato mice, Madisen et al., 2010; Hofer et al., 2011), using either 30 μM Alexa Fluor 594 or Alexa Fluor 488 (Life Technologies, UK) in the internal solution. The targeted cells and patch pipettes were visualized using a custom-built two-photon microscope in the green and red channels with excitation at 880 and 930 nm, respectively.

Data Analysis

All analysis was performed with built-in or custom-made functions in Matlab (MathsWorks, USA). Selectivity index (SI) and mutual information (MI) were calculated as described before (Haider et al., 2010; Borst and Theunissen, 1999) and are explained in detail in the Supplemental Experimental Procedures.

Moment-to-moment differences in Vm (ΔVm) between RF and RF + surround conditions for each neuron were calculated in frame-wide bins (33 ms, Figures 3D and 3I) or 1 ms bins (Figures 3E and 3J) from spike-removed traces (spikes removed at spike threshold, see below). The mean ΔVm during either surround stimulation for each frame was plotted either against the mean Vm relative to spiking threshold (5 mV binning) or against the relative time before firing a spike (−500 ms to −1 ms in 50 ms bins) during the RF stimulation. Spike threshold was determined as in Haider et al. (2010). The membrane potential preceding a spike was first identified, and the membrane potential value at which the second derivative of the membrane potential was maximal was defined as threshold.

Analysis of depolarizing events was carried out by quantifying the number and size of transient positive membrane deflections. Events were detected with a moving window (bin width 5 ms) with an amplitude threshold of 3 mV. An individual event was regarded to have triggered a spike if the peak amplitude of the event was followed by an action potential.

Statistical significance for repeated measurements of the same cell with different stimuli was assessed using the paired Student's t test and ANOVA for reaped measurements (parametric data) or Wilcoxon sign-rank test and Friedman's test (nonparametric data).

SUPPLEMENTAL INFORMATION

Supplemental Information includes six figures and Supplemental Experimental Procedures and can be found with this article at http://dx.doi.org/10.1016/j.neuron.2014.09.010.

AUTHOR CONTRIBUTIONS

M.P. and T.D.M.-F. conceived of the experiments and wrote the paper. E.S., Y.H., and M.P. collected while M.P. and Y.H. analyzed the data.

ACKNOWLEDGMENTS

We are grateful to Dr. N.A. Lesica for help on stimulus design and data analysis. We thank J.A. Movshon, S.L. Smith, B. Haider, S.B. Hofer, N.A. Lesica, and F. Iacaruso for helpful suggestions on different versions of the manuscript. This work was supported by the Humboldt-Foundation (M.P.), the European Research Council, European FP7 Consortium grant ("EuroV1Sion"), and the Wellcome Trust (all T.D.M.-F.).

Accepted: August 22, 2014
Published: September 25, 2014

REFERENCES

Adesnik, H., Bruns, W., Taniguchi, H., Huang, Z.J., and Scanziani, M. (2012). A neural circuit for spatial summation in visual cortex. Nature *490*, 226–231.

Allman, J., Miezin, F., and McGuinness, E. (1985). Stimulus specific responses from beyond the classical receptive field: neurophysiological mechanisms for local-global comparisons in visual neurons. Annu. Rev. Neurosci. *8*, 407–430.

Angelucci, A., and Bressloff, P.C. (2006). Contribution of feedforward, lateral and feedback connections to the classical receptive field center and extra-classical receptive field surround of primate V1 neurons. Prog. Brain Res. *154*, 93–120.

Azouz, R., and Gray, C.M. (2003). Adaptive coincidence detection and dynamic gain control in visual cortical neurons in vivo. Neuron *37*, 513–523.

Barlow, H. (1961). Possible principles underlying the transformation of sensory messages. In Sensory Communication, W. Rosenblith, ed. (Cambridge, MA: MIT Press), pp. 217–234.

Berkes, P., Orbán, G., Lengyel, M., and Fiser, J. (2011). Spontaneous cortical activity reveals hallmarks of an optimal internal model of the environment. Science *331*, 83–87.

Blakemore, C., and Tobin, E.A. (1972). Lateral inhibition between orientation detectors in the cat's visual cortex. Exp. Brain Res. *15*, 439–440.

Blakemore, C., and Van Sluyters, R.C. (1975). Innate and environmental factors in the development of the kitten's visual cortex. J. Physiol. *248*, 663–716.

Borst, A., and Theunissen, F.E. (1999). Information theory and neural coding. Nat. Neurosci. *2*, 947–957.

Chapman, B., and Stryker, M.P. (1993). Development of orientation selectivity in ferret visual cortex and effects of deprivation. J. Neurosci. *13*, 5251–5262.

Chattopadhyaya, B., Di Cristo, G., Higashiyama, H., Knott, G.W., Kuhlman, S.J., Welker, E., and Huang, Z.J. (2004). Experience and activity-dependent maturation of perisomatic GABAergic innervation in primary visual cortex during a postnatal critical period. J. Neurosci. *24*, 9598–9611.

David, S.V., Vinje, W.E., and Gallant, J.L. (2004). Natural stimulus statistics alter the receptive field structure of v1 neurons. J. Neurosci. *24*, 6991–7006.

Espinosa, J.S., and Stryker, M.P. (2012). Development and plasticity of the primary visual cortex. Neuron *75*, 230–249.

Fagiolini, M., Pizzorusso, T., Berardi, N., Domenici, L., and Maffei, L. (1994). Functional postnatal development of the rat primary visual cortex and the role of visual experience: dark rearing and monocular deprivation. Vision Res. *34*, 709–720.

Felsen, G., Touryan, J., and Dan, Y. (2005a). Contextual modulation of orientation tuning contributes to efficient processing of natural stimuli. Network *16*, 139–149.

Felsen, G., Touryan, J., Han, F., and Dan, Y. (2005b). Cortical sensitivity to visual features in natural scenes. PLoS Biol. *3*, e342.

Frégnac, Y., and Imbert, M. (1984). Development of neuronal selectivity in primary visual cortex of cat. Physiol. Rev. *64*, 325–434.

Froudarakis, E., Berens, P., Ecker, A.S., Cotton, R.J., Sinz, F.H., Yatsenko, D., Saggau, P., Bethge, M., and Tolias, A.S. (2014). Population code in mouse V1 facilitates readout of natural scenes through increased sparseness. Nat. Neurosci. *7*, 851–857.

Gilbert, C.D., and Wiesel, T.N. (1990). The influence of contextual stimuli on the orientation selectivity of cells in primary visual cortex of the cat. Vision Res. *30*, 1689–1701.

Girardin, C.C., and Martin, K.A.C. (2009). Inactivation of lateral connections in cat area 17. Eur. J. Neurosci. *29*, 2092–2102.

Guo, K., Robertson, R.G., Mahmoodi, S., and Young, M.P. (2005). Centre-surround interactions in response to natural scene stimulation in the primary visual cortex. Eur. J. Neurosci. *21*, 536–548.

Haider, B., Krause, M.R., Duque, A., Yu, Y., Touryan, J., Mazer, J.A., and McCormick, D.A. (2010). Synaptic and network mechanisms of sparse and reliable visual cortical activity during nonclassical receptive field stimulation. Neuron *65*, 107–121.

Haider, B., Häusser, M., and Carandini, M. (2013). Inhibition dominates sensory responses in the awake cortex. Nature *493*, 97–100.

Hofer, S.B., Ko, H., Pichler, B., Vogelstein, J., Ros, H., Zeng, H., Lein, E., Lesica, N.A., and Mrsic-Flogel, T.D. (2011). Differential connectivity and response dynamics of excitatory and inhibitory neurons in visual cortex. Nat. Neurosci. *14*, 1045–1052.

Hubel, D.H., and Wiesel, T.N. (1963). Receptive fields of cells in striate cortex of very young, visually inexperienced kittens. J. Neurophysiol. *26*, 994–1002.

Iwai, Y., Fagiolini, M., Obata, K., and Hensch, T.K. (2003). Rapid critical period induction by tonic inhibition in visual cortex. J. Neurosci. *23*, 6695–6702.

Jones, H.E., Grieve, K.L., Wang, W., and Sillito, A.M. (2001). Surround suppression in primate V1. J. Neurophysiol. *86*, 2011–2028.

Jones, H.E., Wang, W., and Sillito, A.M. (2002). Spatial organization and magnitude of orientation contrast interactions in primate V1. J. Neurophysiol. *88*, 2796–2808.

Katagiri, H., Fagiolini, M., and Hensch, T.K. (2007). Optimization of somatic inhibition at critical period onset in mouse visual cortex. Neuron *53*, 805–812.

Knierim, J.J., and van Essen, D.C. (1992). Neuronal responses to static texture patterns in area V1 of the alert macaque monkey. J. Neurophysiol. *67*, 961–980.

Ko, H., Cossell, L., Baragli, C., Antolik, J., Clopath, C., Hofer, S.B., and Mrsic-Flogel, T.D. (2013). The emergence of functional microcircuits in visual cortex. Nature *496*, 96–100.

Krug, K., Akerman, C.J., and Thompson, I.D. (2001). Responses of neurons in neonatal cortex and thalamus to patterned visual stimulation through the naturally closed lids. J. Neurophysiol. *85*, 1436–1443.

Kuhlman, S.J., Tring, E., and Trachtenberg, J.T. (2011). Fast-spiking interneurons have an initial orientation bias that is lost with vision. Nat. Neurosci. *14*, 1121–1123.

Laughlin, S.B. (2001). Energy as a constraint on the coding and processing of sensory information. Curr. Opin. Neurobiol. *11*, 475–480.

Lehky, S.R., Sejnowski, T.J., and Desimone, R. (2005). Selectivity and sparseness in the responses of striate complex cells. Vision Res. *45*, 57–73.

Madisen, L., Zwingman, T.A., Sunkin, S.M., Oh, S.W., Zariwala, H.A., Gu, H., Ng, L.L., Palmiter, R.D., Hawrylycz, M.J., Jones, A.R., et al. (2010). A robust and high-throughput Cre reporting and characterization system for the whole mouse brain. Nat. Neurosci. *13*, 133–140.

Maffei, A., Nelson, S.B., and Turrigiano, G.G. (2004). Selective reconfiguration of layer 4 visual cortical circuitry by visual deprivation. Nat. Neurosci. *7*, 1353–1359.

Meyer, A.H., Katona, I., Blatow, M., Rozov, A., and Monyer, H. (2002). In vivo labeling of parvalbumin-positive interneurons and analysis of electrical coupling in identified neurons. J. Neurosci. *22*, 7055–7064.

Nelson, J.I., and Frost, B.J. (1978). Orientation-selective inhibition from beyond the classic visual receptive field. Brain Res. *139*, 359–365.

Nienborg, H., Hasenstaub, A., Nauhaus, I., Taniguchi, H., Huang, Z.J., and Callaway, E.M. (2013). Contrast dependence and differential contributions from somatostatin- and parvalbumin-expressing neurons to spatial integration in mouse V1. J. Neurosci. *33*, 11145–11154.

Oliva, A.A., Jr., Jiang, M., Lam, T., Smith, K.L., and Swann, J.W. (2000). Novel hippocampal interneuronal subtypes identified using transgenic mice that express green fluorescent protein in GABAergic interneurons. J. Neurosci. *20*, 3354–3368.

Olshausen, B.A., and Field, D.J. (1996). Emergence of simple-cell receptive field properties by learning a sparse code for natural images. Nature *381*, 607–609.

Olshausen, B.A., and Field, D.J. (2004). Sparse coding of sensory inputs. Curr. Opin. Neurobiol. *14*, 481–487.

Olveczky, B.P., Baccus, S.A., and Meister, M. (2003). Segregation of object and background motion in the retina. Nature *423*, 401–408.

Ozeki, H., Finn, I.M., Schaffer, E.S., Miller, K.D., and Ferster, D. (2009). Inhibitory stabilization of the cortical network underlies visual surround suppression. Neuron *62*, 578–592.

Rochefort, N.L., Narushima, M., Grienberger, C., Marandi, N., Hill, D.N., and Konnerth, A. (2011). Development of direction selectivity in mouse cortical neurons. Neuron *71*, 425–432.

Ruthazer, E.S., and Stryker, M.P. (1996). The role of activity in the development of long-range horizontal connections in area 17 of the ferret. J. Neurosci. *16*, 7253–7269.

Sachdev, R.N.S., Krause, M.R., and Mazer, J.A. (2012). Surround suppression and sparse coding in visual and barrel cortices. Front. Neural Circuits *6*, 43.

Self, M.W., Lorteije, J.A., Vangeneugden, J., van Beest, E.H., Grigore, M.E., Levelt, C.N., Heimel, J.A., and Roelfsema, P.R. (2014). Orientation-tuned surround suppression in mouse visual cortex. J. Neurosci. *34*, 9290–9304.

Seriès, P., Lorenceau, J., and Frégnac, Y. (2003). The "silent" surround of V1 receptive fields: theory and experiments. J. Physiol. Paris *97*, 453–474.

Shen, Z.-M., Xu, W.-F., and Li, C.-Y. (2007). Cue-invariant detection of centre-surround discontinuity by V1 neurons in awake macaque monkey. J. Physiol. *583*, 581–592.

Simoncelli, E.P., and Olshausen, B.A. (2001). Natural image statistics and neural representation. Annu. Rev. Neurosci. *24*, 1193–1216.

Solomon, S.G., Lee, B.B., and Sun, H. (2006). Suppressive surrounds and contrast gain in magnocellular-pathway retinal ganglion cells of macaque. J. Neurosci. *26*, 8715–8726.

Stettler, D.D., Das, A., Bennett, J., and Gilbert, C.D. (2002). Lateral connectivity and contextual interactions in macaque primary visual cortex. Neuron *36*, 739–750.

Tolhurst, D.J., Smyth, D., and Thompson, I.D. (2009). The sparseness of neuronal responses in ferret primary visual cortex. J. Neurosci. *29*, 2355–2370.

Vaiceliunaite, A., Erisken, S., Franzen, F., Katzner, S., and Busse, L. (2013). Spatial integration in mouse primary visual cortex. J. Neurophysiol. *110*, 964–972.

Vinje, W.E., and Gallant, J.L. (2000). Sparse coding and decorrelation in primary visual cortex during natural vision. Science *287*, 1273–1276.

Vinje, W.E., and Gallant, J.L. (2002). Natural stimulation of the nonclassical receptive field increases information transmission efficiency in V1. J. Neurosci. *22*, 2904–2915.

White, L.E., Coppola, D.M., and Fitzpatrick, D. (2001). The contribution of sensory experience to the maturation of orientation selectivity in ferret visual cortex. Nature *411*, 1049–1052.

Willmore, B.D.B., Mazer, J.A., and Gallant, J.L. (2011). Sparse coding in striate and extrastriate visual cortex. J. Neurophysiol. *105*, 2907–2919.

Xu, W.-F., Shen, Z.-M., and Li, C.-Y. (2005). Spatial phase sensitivity of V1 neurons in alert monkey. Cereb. Cortex *15*, 1697–1702.

Zufferey, P.D., Jin, F., Nakamura, H., Tettoni, L., and Innocenti, G.M. (1999). The role of pattern vision in the development of cortico-cortical connections. Eur. J. Neurosci. *11*, 2669–2688.

Neuron, Volume *84*

Supplemental Information

Experience-Dependent Specialization of Receptive Field Surround for Selective Coding of Natural Scenes

Michael Pecka, Yunyun Han, Elie Sader, and Thomas D. Mrsic-Flogel

Supplemental Information

Experience-dependent specialization of receptive field surround for selective coding of natural scenes

Michael Pecka, Yunyun Han, Elie Sader, and Thomas D. Mrsic-Flogel

Supplemental Inventory

Six supplemental figures with legends and supplemental Experimental Procedures:

Figure S1 relates to figure 1
Figure S2 relates to figure 2
Figure S3 relates to figures 3 and 4
Figure S4 relates to figure 5
Figure S5 relates to figures 3, 5 and 6
Figure S6 relates to figures 2 and 6

Supplemental Experimental Procedures:
Description of procedures and analyses used for data presented in figures 1 – 6 and S1 – S6.

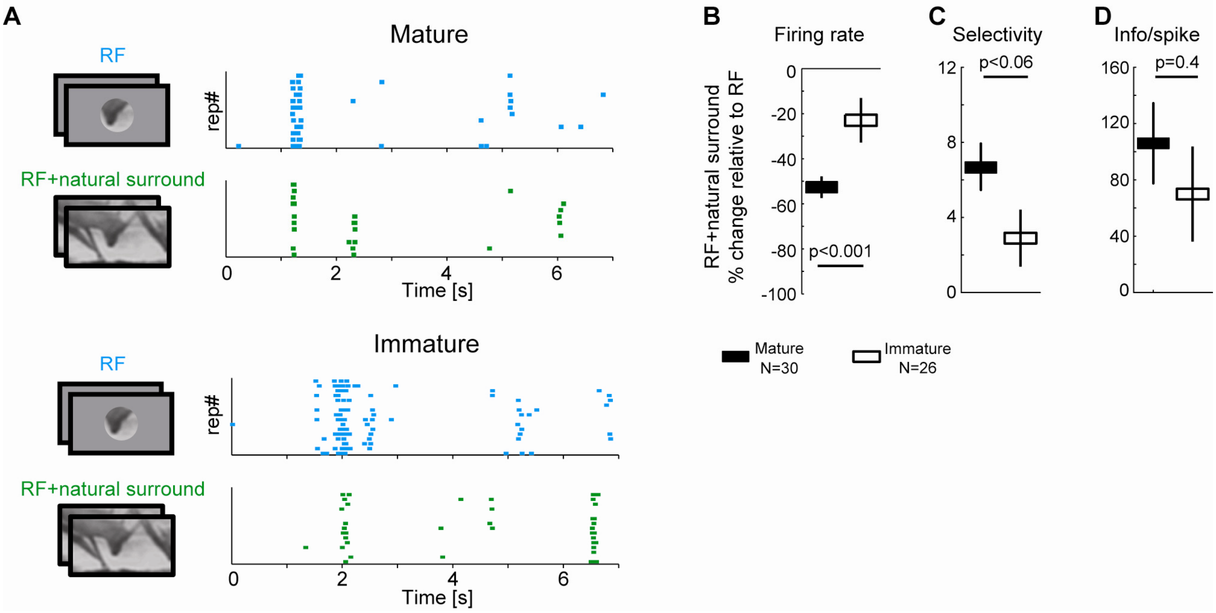

Figure S1. Surround modulation in mature and immature mice

(A) Raster plot display of an example cell from a mature (upper two panels) and immature mouse (lower two panels) recorded in juxta-cellular mode. Similar to the results from whole-cell recordings, co-stimulation of the surround with natural stimuli lead to response suppression in both age-groups.

(B)-(D) Quantifications of changes in firing rate (B), selectivity (C) and info/spike (D) for all cells recorded in juxta-cellular mode. Similar to the results from whole-cell recordings, significant decrease in firing rate and increased selectivity and information transmitted per spike were found for co-stimulation of the surround in either age-group, yet changes were stronger in mature animals than in immature animals. Conventions are as in Figure 1.

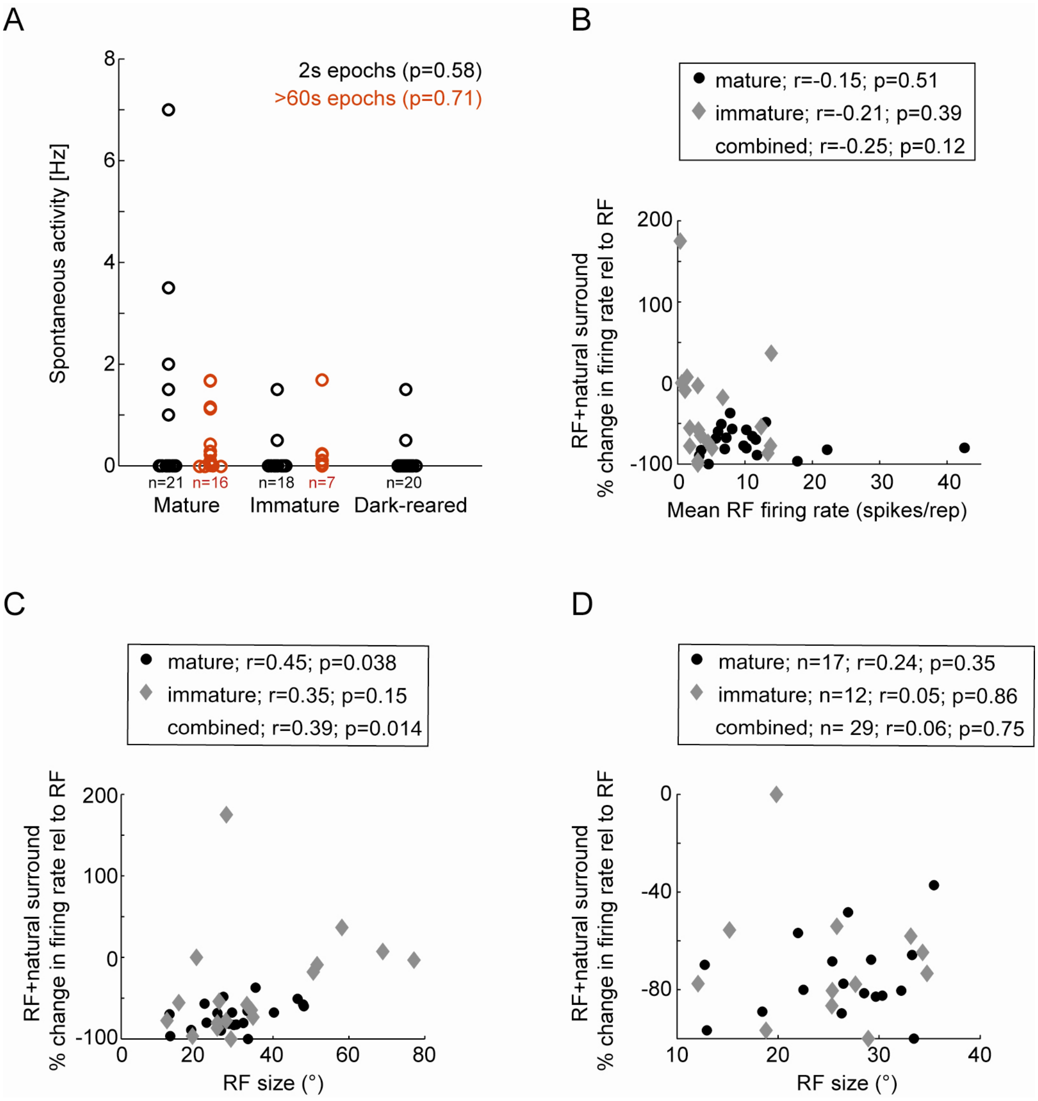

Figure S2. Surround suppression is not affected by levels of spontaneous and evoked activity or RF size.

(A) Spontaneous activity in the population of neurons (black) was measured using 2s long epochs of gray screen presentation prior to visual stimulation. Most cells exhibited negligible spontaneous rates<1Hz. Spontaneous rates were not different between age-groups (p=0.58; Friedman's test). In a separate population of cells (red), these low levels of spontaneous activity were confirmed by using long epochs (>60s) of gray screen presentation (p=0.71; Wilcoxon rank sum test).
(B) Scatter plot of firing rate suppression during RF+natural surround stimulation and the mean firing rate during RF stimulation for all neurons in mature (black filled circles) and immature (gray diamonds) mice. The level of suppression and mean RF firing rate were not correlated (Spearson's correlation and corresponding p-values are given in box above panel).
(C)-(D) Scatter plot of firing rate suppression during RF+natural surround stimulation and the RF size. Conventions as in (B). The level of suppression was mildly correlated with the RF size for data from mature mice and when combining immature and mature data. However, the correlation was predominantly caused by a few neurons woth very large RF sizes, as no correlations was found for neurons with RF sizes <40° (D, number of neurons given in box above panel).

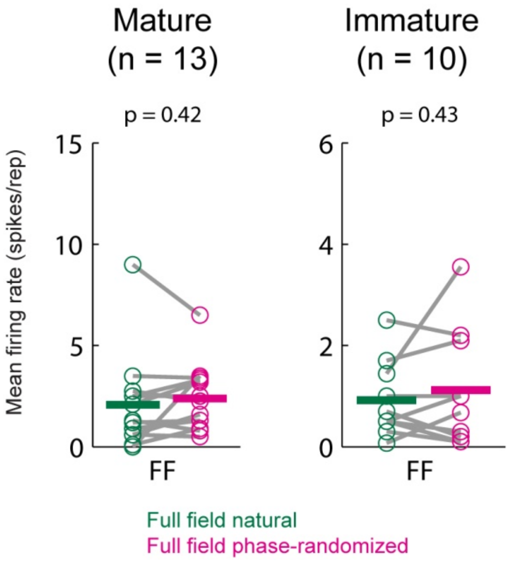

Figure S3. Full field presentation of phase-randomized movies evokes similar activity levels as natural movies.

Firing rates of Pyramidal neurons in mature (left) and immature (right) V1 were not significantly different between full field presentations of natural and phase-randomized movies. P-values derived from paired t-tests.

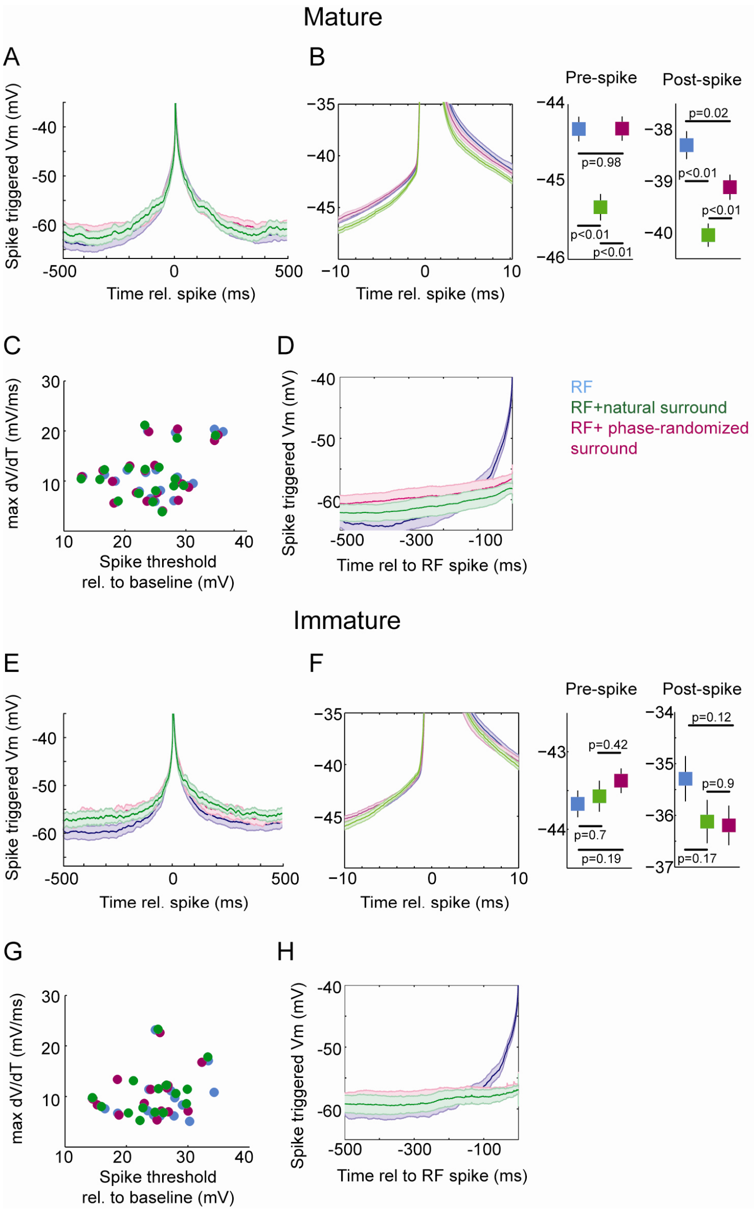

Figure S4. Average Vm prior to spiking reveals increased hyperpolarization during natural surround stimulation in mature V1.

(A) Spike-triggered averages (STAs) for all three stimulus conditions ±500ms depiciting the mean Vm (±S.E.M.) of neurons from mature mice (n=21) relative to the occurrence of a spike (see Supplemental Methods for details).
(B) Left-hand panel shows zoom-in from A) at ±10ms. RF+natural surround was significantly more hyperpolarized in the last 10ms before spiking (quantifications are shown in right-hand panels, p-values derived from t-tests)
(C) Spike threshold and maximal dV/dt did not differ across stimulus conditions.
(D) RF-STAs for the three stimulus conditions (see Supplemental Methods for details) reveal a stronger hyperpolarization during RF+natural surround compared to RF+phase-randomized surround stimulation.
(E)-(H) Same as in (A)-(D) for immature mice (n=18). In contrast to mature mice, no significant differences in hyperpolarization before spiking were present.

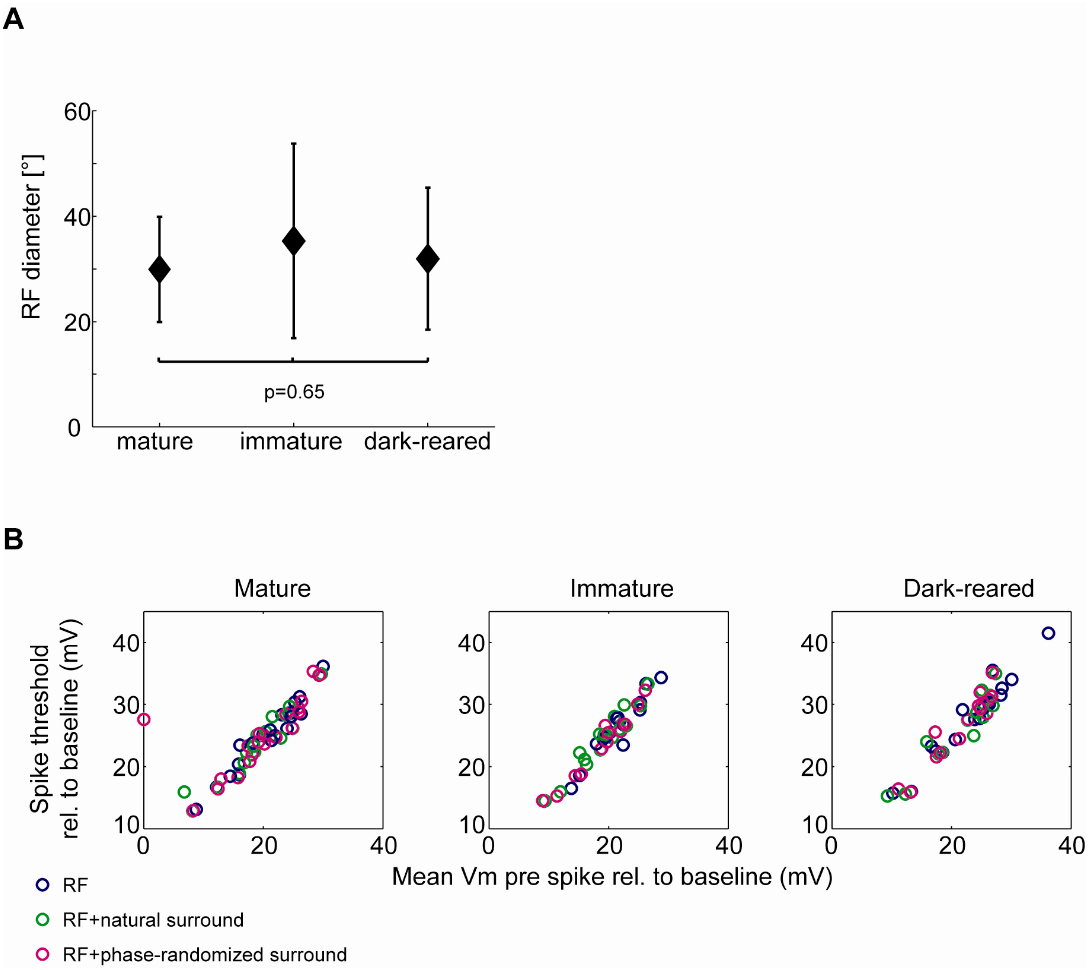

Figure S5. RF sizes and membrane properties were similar for all age-groups

(A) Mean RF diameter (+-S.E.M.) were similar in the three age-groups tested (n=21, 18, 19 for mature, immature and dark-reared, respectively; P=0.65, one-way ANOVA).
(B) Spike threshold of individual cells (open circles) is plotted as a function of the mean Vm within 10ms preceding the spike. The relationship was highly consistent both across stimulus condition and age-group.

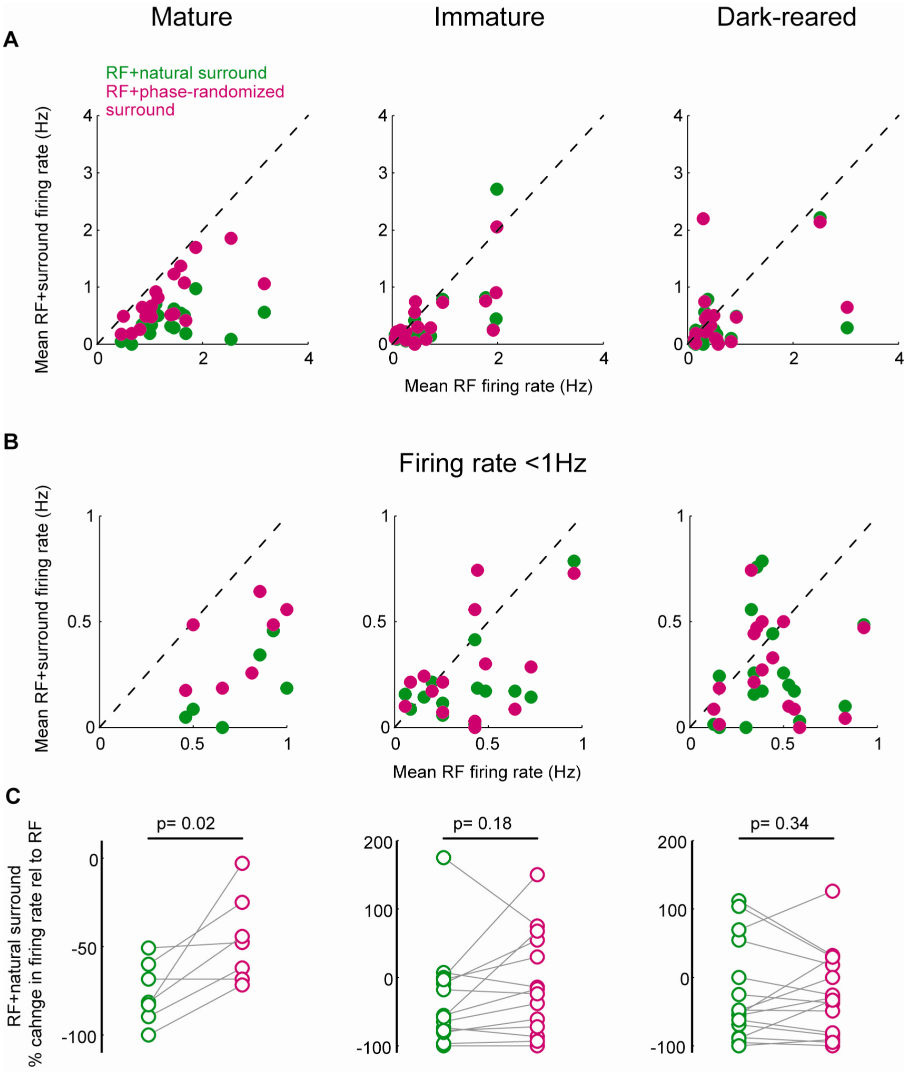

Figure S6. The level of firing rate suppression is not related to absolute firing rates.

(A) Scatter plot displays of mean RF firing rates and mean RF+surround firing rates for all neurons in the three age groups. Clearly, response suppression is most prominent in mature mice, particularly for natural surround stimulation.
(B) Scatter plots as in A) for the sub-population of cells with firing rates < 1Hz, demonstrating that the more suppressive effect of RF+surround stimulation in mature mice compared to the other age groups is not related to the overall lower firing rates in immature and dark-reared mice.
(C) Pair-wise analysis of neurons with firing rates < 1Hz confirms that absolute firing rates do not affect the sensitivity of surround suppression for natural stimuli. P-values are derived from paired t-tests.

Supplemental Experimental Procedures:

Surgery. To prevent dehydration of the cornea, a thin layer of cream (Isoptomax) was applied to the eyes before it was carefully removed prior to the start of the recordings. Animals were placed on a heating pad to maintain constant body temperature. Atropine (0.01 ml at 0.1 mg/ml) and Fortecortin (0.01 ml at 2 mg/ml) were injected subcutaneously. The skull was immobilized by affixing it to a metal plate with dental cement. A small (~1mm) craniotomy was performed above the monocular region of the visual cortex, as determined by stereotactic coordinates, and later confirmed with retinotopic mapping. A durectomy was occasionally performed to improve the quality of the recordings. Dehydration of the exposed cortical surface was prevented by regular administration of cortex buffer (150 mM NaCl, 2.5 mM KCl, 10 mM HEPES, pH 7.4). The eye ipsilateral to the craniotomy was covered to prevent any binocular stimulation.

Stimuli. Stimuli were presented to the contralateral eye on a 30'' TFT monitor (Samsung SyncMaster 305T) positioned at 27cm distance to the animal's head. The monitor was centered on the RF of the recorded neuron if possible. The frame rate was set to 30 Hz and the monitor refresh rate was set to 60 Hz. A naturalistic movie was presented consisting of multiple sequences of continuous naturalistic scenes (each typically ~1000ms in duration) with cross-fading of individual sequences (sequences were taken from David Attenborough's Life of Mammals, BBC). The resolution of the movie was set to 100 x 56 pixels (horizontal x vertical) and spatial frequencies of the movie were low-pass filtered at 0.16 cycles/degree to account for the spatial acuity of mice (Niell and Stryker, 2008), thereby maximizing the contrast of the movie within the spectrum that mice are able to detect. The mean contrast of the naturalistic movie and phase-randomized noise movies over all frames was set to 0.7. Phase-randomized movies were generated by randomizing the phases of the spatial frequency spectrum of the natural movie. Phase-randomized movies were subsequently low-pass filtered at 0.16 cycles/degree and the mean luminance and mean contrast was set to match the original naturalistic movie.

Electrophysiology and data acquisition. Pipettes were advanced into the cortex at 40o angle with a high positive pressure until the electrode tip was at the depth of approximately 100 µm (corresponding to superficial layer 2/3). The positive pressure was then reduced to 20-40 mmHg and the pipette was advanced in steps of 2.5 µm until the pipette resistance increased suddenly and a bouncy baseline detected – indicating contact with a cell. At this point the pressure was released, and slight suction was subsequently applied until a gigaseal was formed, and then the whole-cell configuration was established by applying quick steps of negative pressure. Capacitance compensation and bridge balance were monitored and carefully adjusted after obtaining the gigaseal. Except for brief current pulses to assess firing pattern, no current was injected during then recording and the membrane potential was not corrected for liquid junction potentials.

Data analysis. We calculated the lifetime response selectivity index (SI) as previously described (Vinje and Gallant, 2000; Haider et al., 2010):
$SI=\{1-[(\sum r_i/n)^2/\sum(r_i^2/n)]\}/[1-(1/n)]$,
where r_i is the average response to the ith frame of the movie, and n is the number of movie frames. The resulting values of SI are bounded between 0 and 1. A value of 1 indicates responses to only one frame in all repetitions, while a value of 0 indicates completely

random or unselective firing to any frame. Importantly, SI has a sigmoid shape as a function of frame selectivity with asymptotic behavior near 0 and 1. In our dataset, SI was generally rather high (typically > 0.8), hence significant changes in selectivity resulted in only modest relative changes in SI.

Information transmission was measured by using a direct method to calculate the average mutual information (Shannon, 1948) as: MI= H(R) - H(R|S), where H(R) is the spiking response entropy and H(R|S) is the noise entropy. Spike trains were divided into 15 ms time windows to obtain binary response trains on which noise and response entropies were calculated as described previously (Borst and Theunissen, 1999; Magri et al., 2009). Time windows of 5 ms, 33 ms (movie frame duration) and 60 ms were also tested and yielded qualitatively similar results. To compensate for any overestimation of MI caused by the limited number of trails, we applied the analytical Panzeri-Treves bias correction (Panzeri and Treves, 1996; Magri et al., 2009). A shuffling bias correction procedure was additionally performed (Panzeri et al 2007), but did not further influence the results. To determine the significance of the calculated information for each neuron, 100 repetitions of MI calculations with randomized binary response trains were performed using bootstrapping methods (Magri et al., 2009). A neuron was only considered for further analysis if MI of the original spike train exceeded the mean MI +2*S.D. of the randomized spike trains. Values of MI were divided by the respective number of spikes to give the information for each spike (information/spike) (Vinje and Gallant, 2002). For comparisons of firing rate, SI and mutual information/spike across age-groups (Fig. 2), analysis was restricted to cells that exhibited significant firing rate suppression in response to at least one surround condition.

Spike-triggered averages (STA) were computed by averaging the membrane potential recordings for ±500ms relative to the peak voltage deflection of a spike. To eliminate confounding influences on the mean Vm, voltage traces were only included in this analysis if spikes were not preceded or followed by another spike within +-500ms. For RF-STAs, time periods were determined based on the occurrences of spikes during the RF condition only. The Vm during RF+surround stimulation were analyzed for the equivalent time periods as in the RF condition regardless whether spikes had occurred in the RF+surround condition.

Supplemental References:

Magri, C., Whittingstall, K., Singh, V., Logothetis, N.K., and Panzeri, S. (2009). A toolbox for the fast information analysis of multiple-site LFP, EEG and spike train recordings. BMC Neurosci 10, 81.

Panzeri, S., and Treves, A. (1996). Analytical estimates of limited sampling biases in different information measures. Network: Computation in Neural Systems 7, 87–107.

Panzeri, S., Senatore, R., Montemurro, M.A., and Petersen, R.S. (2007). Correcting for the Sampling Bias Problem in Spike Train Information Measures. J Neurophysiol 98, 1064–1072.

Shannon, C.J. (1948). A mathematical theory of communication. Bell Systems Techincal Journal 27, 379–423.
